# Microplastic contamination in agricultural systems: impacts on plant productivity, economic losses, and supply chain resilience

**DOI:** 10.3389/fpls.2026.1956066

**Published:** 2026-10-09

**Authors:** Tong Xu

**Affiliations:** College of Economics, Hunan Agriculture University, Changsha, Hunan, China

**Keywords:** agricultural soils, crop productivity, economic losses, food security, microplastics, nanoplastics, phytotoxicity, plasticulture

## Abstract

Microplastics (MPs) have transitioned from a predominantly marine concern to a defining threat facing terrestrial agroecosystems, where croplands now function as one of the largest global reservoirs of plastic particles. This review synthesizes evidence to construct an integrated narrative that links the entry of MPs into farmland with declining plant productivity, quantifiable economic losses, and the erosion of agricultural supply chain resilience. The synthesis is based on a documented search of Web of Science, Scopus, ScienceDirect and Google Scholar covering January 2018 to March 2026, with explicit inclusion and exclusion criteria reported in Section 1.1. Sources, fate and soil-phase processes are treated in condensed form, because they are already covered comprehensively elsewhere; we retain only the evidence that propagates into crop, economic and supply-chain outcomes. Building on these soil-level disturbances, we evaluate the phytotoxic consequences for crops, spanning inhibited germination, impaired root architecture, reduced photosynthetic efficiency, disrupted nutrient acquisition, and the uptake and translocation of nano-sized particles into edible tissues. A central contribution of this review is the explicit coupling of these biophysical impacts to their economic and systemic dimensions, because recent global meta-analyses estimate that MP-induced photosynthetic suppression alone may reduce staple crop production by tens to hundreds of millions of tons annually, translating into substantial revenue losses, quality downgrading, remediation expenditure, and heightened exposure of food supply chains to shocks. Rather than reporting these modelled outputs at face value, we dissect the inferential chain that produces them, from effect size to biomass to harvestable yield to price, and we identify the assumptions at each step that determine whether the resulting figures are decision-grade. We complement the global estimates with farm-level cost–benefit evidence showing that the private returns to plasticulture remain positive while the contamination externality contributes under one percent of the accounted benefits of current management, which explains the persistence of practices that degrade the productive base. Throughout, illustrative case studies from China, Chile, Australia, and Europe are embedded within each thematic section rather than treated in isolation, demonstrating how local contamination patterns propagate into landscape- and market-scale consequences. We further appraise mitigation and remediation options, ranging from source reduction and material substitution to biochar amendment, and we assess emerging governance instruments such as the FAO Voluntary Code of Conduct and circular-economy regulation. Finally, we identify persistent knowledge gaps concerning the calibration of field-realistic dose–response relationships, the construction of decision-grade economic estimates, and the severe geographical imbalance of the evidence base, in which South Asia, sub-Saharan Africa and Latin America are collectively represented by a small fraction of published field studies, and we outline research priorities to safeguard soil health and food security. The review argues that microplastic contamination should be reframed not merely as an ecotoxicological problem but as a slow-onset stressor with strategic implications for the stability of global food systems.

## 1 Introduction

Plastic has become one of the most consequential materials of the modern era, and its production has expanded from roughly two million tons in the 1950s to well over four hundred million tons annually, with output projected to reach approximately five hundred million tons by the middle of the present decade (Geyer et al., 2017; MacLeod et al., 2021). Only a small fraction of this material is recycled, and the overwhelming majority is landfilled, incinerated, or released into the environment, where it fragments into microplastics defined as particles smaller than five millimeters (Thompson et al., 2004; Cole et al., 2011). While early scientific attention focused almost exclusively on oceans, it is now firmly established that terrestrial systems, and agricultural soils in particular, receive and retain far greater quantities of plastic than marine waters (Nizzetto et al., 2016; Ng et al., 2018). Agricultural soils have consequently been described as long-term sinks for plastic particles, accumulating inputs from mulching, organic amendments, irrigation, and deposition over successive cropping cycles (Ali et al., 2026; Sattar et al., 2025; Kumar et al., 2020). This accumulation is not ecologically neutral, because MPs interact with the physical, chemical, and biological components of soil that jointly determine fertility and crop performance (de Souza Machado et al., 2019; Rillig and Lehmann, 2020). The scale of the problem has prompted international bodies, including the Food and Agriculture Organization, to classify plastic pollution among the foremost environmental risks to food production (FAO, 2025; Ali et al., 2024). Recent global modelling has sharpened these concerns by estimating that microplastic-induced suppression of photosynthesis could reduce the production of wheat, rice, and maize by several percentage points worldwide (Zhu et al., 2025; Ding et al., 2024; Ma et al., 2023). Such findings elevate MPs from a localized soil-quality issue to a matter of strategic significance for global food security (Zhu et al., 2025; Garfansa et al., 2025). The convergence of these lines of evidence signals that microplastic contamination now warrants systematic assessment across the full agricultural value chain. This review responds to that need by integrating soil, plant, economic, and systemic perspectives within a single analytical frame.

The agricultural sector is simultaneously a victim and a vector of plastic pollution, a duality that complicates both diagnosis and response. On one hand, plasticulture practices such as film mulching, greenhouse cultivation, tunnels, and drip-irrigation tubing have delivered demonstrable gains in yield, water-use efficiency, and season extension across diverse climates (Yang et al., 2022; Sattar et al., 2025). On the other hand, the same practices leave persistent residues that fragment into MPs and gradually contaminate the very soils on which production depends (Qi et al., 2018; Huang et al., 2020). This resilience paradox, whereby a short-term productivity aid becomes a long-term productivity liability, lies at the heart of the contemporary debate about sustainable intensification (Gontard, 2025). Compounding the challenge, MPs rarely occur in isolation, instead co-existing with heavy metals, pesticides, antibiotics, and other pollutants that they can adsorb and transport through soil-plant systems (Liu et al., 2024; Ren et al., 2021). The heterogeneity of polymer types, particle sizes, shapes, and additive chemistries further means that the effects of MPs are highly context-dependent and difficult to generalize (Chae and An, 2018; de Souza Machado et al., 2019). Field-realistic studies remain comparatively scarce, and much of the mechanistic evidence derives from laboratory experiments using pristine particles at concentrations that may exceed those typically encountered in the field (Ng et al., 2018; Cai et al., 2023). These methodological tensions have generated genuine scientific disagreement about the magnitude and even the direction of some effects. Nonetheless, the weight of recent evidence points consistently toward net negative consequences for soil health and crop productivity under realistic long-term exposure (Sarfraz et al., 2025; Zhu et al., 2025). Resolving the remaining uncertainties is therefore a priority that this review seeks to advance.

The novelty of the present contribution lies in its deliberate integration of three domains that are usually treated separately in the literature, namely plant productivity, economic losses, and supply chain resilience. Most existing reviews concentrate on soil physicochemical change or phytotoxicity, and comparatively few connect these biophysical outcomes to the financial and systemic consequences that ultimately determine policy attention (Sutanto et al., 2024; Gontard, 2025). By explicitly following the causal chain from particle to plant to profit to system, this review offers a framework that is both mechanistically grounded and decision-relevant. We further depart from convention by embedding case studies within each thematic section rather than confining them to a dedicated block, so that empirical examples continuously illustrate the arguments as they unfold. This structure allows contamination patterns documented in Chinese cotton fields, Chilean sludge-amended plots, Australian biosolid trials, and European floodplains to be read in direct relation to the mechanisms and consequences they exemplify (Yu et al., 2023; Corradini et al., 2019; Adhikari et al., 2024; Scheurer and Bigalke, 2018). The temporal scope is restricted primarily to research published from 2020 onward, ensuring that the synthesis reflects the rapid maturation of the field, while the introduction foregrounds the most recent 2025 and 2026 studies that have reshaped its priorities (Zhu et al., 2025; Yin et al., 2026). The review is organized to move progressively outward from sources and soil processes, through phytotoxicity and productivity, to economics, food safety, systemic resilience, and mitigation. Because the source, fate and soil-process literature has already been synthesized repeatedly and at length, those themes are compressed into a single condensed section that retains only the evidence required to support the downstream analysis, and the analytical weight of the article is placed instead on the productivity, economic and systemic sections where the contribution is genuinely new. Each section closes analytical loops by referencing quantitative data compiled in the accompanying tables and by signposting the figures suggested at the end of the article. In doing so, we aim to provide researchers, agronomists, and policymakers with a coherent evidence base for anticipating and managing an emerging and insufficiently appreciated threat. The overarching thesis is that microplastic contamination behaves as a slow-onset systemic stressor whose cumulative costs are only now becoming visible. Recognizing it as such is the first step toward proportionate and timely intervention.

To orient the reader, it is useful to establish the terminological and conceptual boundaries adopted here at the outset of the discussion. Microplastics are conventionally defined as synthetic polymer particles between one micrometer and five millimeters, while the term nanoplastics denotes particles below one micrometer that exhibit distinct transport and toxicity behavior owing to their capacity to cross biological membranes (Thompson et al., 2004; Wang et al., 2022). Primary microplastics are manufactured at small sizes, whereas secondary microplastics form through the environmental fragmentation of larger items such as mulch films and packaging, and it is the secondary fraction that dominates in agricultural soils (Cole et al., 2011; Yang et al., 2022). The physicochemical identity of a particle, encompassing polymer chemistry, size distribution, morphology, surface charge, and adsorbed additives, governs how it interacts with soil, water, and organisms, which is why generalizations must be made cautiously (Horton et al., 2017; Liu et al., 2024). Agroecosystems are understood here as coupled soil-plant-water-management systems that are simultaneously ecological and economic entities, a framing that justifies the integrated approach of this review (Gontard, 2025). Supply chain resilience is defined as the capacity of the food system to absorb, adapt to, and recover from disturbances, including the slow-onset disturbance represented by chronic contamination (Gontard, 2025). Food security, in the sense used by the FAO, requires stable availability, access, and utilization of safe and nutritious food, all of which MPs can potentially undermine (FAO, 2025; Zhu et al., 2025). These definitions are not merely semantic, because ambiguity in particle characterization has been a persistent source of inconsistency across studies (Rillig, 2012; Ng et al., 2018). By fixing these terms early, the review seeks conceptual clarity for the analysis that follows. The remainder of the article develops the evidence within this shared vocabulary. Where disagreement persists in the primary literature, we present the competing interpretations rather than imposing a false consensus.

### 1.1 Scope, literature search strategy, and eligibility criteria

Because this review advances quantitative claims about contamination magnitudes, yield penalties and monetary losses, the provenance of the underlying evidence must be transparent, and we therefore report the search protocol explicitly rather than leaving it implicit. The literature was identified through structured searches of Web of Science Core Collection, Scopus, ScienceDirect and Google Scholar, with Google Scholar used only for forward and backward citation chasing rather than as a primary index. The search window ran from January 2018 to March 2026, a period chosen because the terrestrial microplastic literature expanded rapidly after 2018 and because earlier work is adequately represented in the foundational sources that we cite for definitional and conceptual purposes. Search strings combined three concept blocks using Boolean operators, namely a contaminant block (“microplastic*” OR “nanoplastic*” OR “plastic residue*” OR “mulch film*”), a system block (“agricultur *soil*” OR farmland OR cropland OR agroecosystem *OR “soil-plant”), and an outcome block (yield OR productivity OR phytotoxicity OR photosynthesis OR “economic loss*” OR cost OR “food safety” OR “supply chain” OR remediation). Retrieved records were screened first by title and abstract and then by full text, and reference lists of all included reviews were hand-searched to capture studies missed by the database queries. Studies were eligible if they were peer-reviewed primary research, meta-analyses, or authoritative institutional reports, were published in English, and reported either measured microplastic abundance in agricultural matrices, an experimentally determined biological response of a crop or soil organism, or an explicit economic or governance analysis of agricultural plastics. Studies were excluded if they addressed exclusively marine, freshwater or sediment compartments without a terrestrial agricultural component, if they reported only analytical method development without accompanying environmental or biological data, if exposure concentrations were reported in a form that could not be interpreted, or if they were conference abstracts, theses or non-peer-reviewed commentary. Reporting followed the principles of the PRISMA 2020 statement adapted to a narrative synthesis, in the sense that identification, screening and eligibility decisions were documented and are reproducible, although no formal quantitative pooling was performed because the heterogeneity of exposure units precludes it (Page et al., 2021). The same approach has recently been adopted by field-data syntheses in this domain, which likewise combined database searching with PRISMA-style screening before extracting comparable observations (Roy et al., 2025). **Table 1** summarizes the protocol in full, and we note where the retrieved corpus is geographically unbalanced, a limitation revisited in Section 8. This documentation is intended to allow readers to judge which of our quantitative statements rest on multiple independent field datasets and which rest on single studies or modelled extrapolation.

**Table 1:** Literature search protocol, eligibility criteria and evidence handling adopted in this review.

Element	Specification adopted
Databases	Web of Science Core Collection; Scopus; ScienceDirect; Google Scholar (citation chasing only)
Search window	January 2018 – March 2026 (foundational pre-2018 sources retained only for definitions and first reports)
Concept block 1 (contaminant)	“microplastic*” OR “nanoplastic*” OR “plastic residue*” OR “mulch film*”
Concept block 2 (system)	“agricultur *soil*” OR farmland OR cropland OR agroecosystem* OR “soil-plant”
Concept block 3 (outcome)	yield OR productivity OR phytotoxicity OR photosynthesis OR “economic loss*” OR cost OR “food safety” OR “supply chain” OR remediation
Inclusion criteria	Peer-reviewed primary studies, meta-analyses or authoritative institutional reports in English reporting (i) measured MP abundance in agricultural matrices, (ii) an experimentally determined crop or soil-organism response, or (iii) an explicit economic, policy or governance analysis
Exclusion criteria	Exclusively marine/freshwater/sediment studies; method-development papers without environmental or biological data; uninterpretable exposure reporting; conference abstracts, theses and non-peer-reviewed commentary
Screening	Title/abstract screening followed by full-text assessment; backward and forward citation chasing of all included reviews
Synthesis approach	Narrative synthesis following PRISMA 2020 reporting principles (Page et al., 2021); no quantitative pooling, because exposure units (particles kg^−^¹ vs. % w/w) are not interconvertible
Known corpus bias	Strong over-representation of East Asian, and particularly Chinese, field data; sparse coverage of South Asia, sub-Saharan Africa and Latin America (see Section 8)

### 1.2 Relationship to previous reviews and the specific contribution of this synthesis

A review of an actively synthesized field must state plainly what previous syntheses have already achieved and what remains for it to add, and we therefore close the introduction by positioning this article explicitly against its predecessors. The first generation of terrestrial microplastic reviews established the problem and mapped the sources, showing that agricultural soils receive and retain more plastic than the oceans and identifying mulching, amendments, irrigation and deposition as the dominant pathways (Ng et al., 2018; Kumar et al., 2020). A second generation consolidated the soil-phase evidence, describing fragmentation kinetics, vertical transport, aggregate-scale distribution and the plastisphere as a distinct microbial habitat, and these questions can now be regarded as comprehensively covered (Qi et al., 2020; Cai et al., 2023; Rillig et al., 2024). A third and largely contemporaneous group has synthesized ecotoxicological outcomes for plants and soil biota, culminating in quantitative meta-analyses that report trait-level effect sizes across crops and polymers (Sun H. et al., 2024; Sun X. et al., 2024). Parallel work has extended the frame to fate, transport and governance across the whole soil-to-human continuum, and to source-resolved risk matrices linking specific on-farm practices to particle traits (Boctor et al., 2025; Roy et al., 2025; Hoang et al., 2024). What none of these syntheses does is carry the biophysical signal through to a monetary and systemic account, and the few papers that do monetize losses either report modelled outputs without interrogating their construction or examine a single crop set in isolation (Sutanto et al., 2024; Zhu et al., 2025). The distinct contribution of the present review is therefore threefold. First, it dissects rather than reports the inferential chain by which photosynthetic and morphological effect sizes are converted into yield, revenue and welfare estimates, identifying the specific assumptions that make current global figures indicative rather than decision-grade. Second, it places those top-down estimates alongside bottom-up farm-level cost–benefit evidence, showing that the private profitability of plasticulture and the social cost of contamination are accounted in entirely different ledgers (Ma et al., 2018; Hao et al., 2024; Velandia et al., 2020a). Third, it treats the food system rather than the field as the unit of analysis, asking how a slow-onset, spatially correlated stressor propagates through concentrated production regions and traded staple markets. **Table 2** sets out this positioning against representative prior reviews, and the remainder of the article is weighted accordingly, with the established source-and-fate material compressed into Section 2 and the analytical emphasis placed on Sections 3 to 7.

**Table 2:** Positioning of the present review relative to representative prior syntheses on microplastics in agricultural systems.

Review	Sources & fate	Soil processes	Crop phytotoxicity	Monetized economics	Supply-chain/systemic analysis	Principal focus
Ng et al. (2018)	●	◐	◐	○	○	First mapping of MP/NP pollution in agroecosystems
Kumar et al. (2020)	●	●	◐	○	○	MPs as soil pollutants; sources and behavior
Qi et al. (2020)	●	●	◐	○	○	Film residues and MP behavior in soil
Cai et al. (2023)	●	●	○	○	○	Abundance and distribution in Chinese farmland
Hoang et al. (2024)	●	●	◐	○	○	Comprehensive sources, fate and impacts
Sun X. et al. (2024)	◐	◐	●	○	○	Critical review of impacts on terrestrial plants
Sun H. et al. (2024)	○	●	●	○	○	Global meta-analysis of soil and crop trait responses
Boctor et al. (2025)	●	●	◐	○	◐	Fate, transport and governance to food webs and humans
Roy et al. (2025)	●	◐	◐	○	◐	Source-to-risk matrix from 105 field datasets
Sutanto et al. (2024)	○	○	◐	●	○	Yield and economic loss for five commodities
Gontard (2025)	◐	○	○	◐	●	Plastic particle footprint of the agri-food sector
This review	◐	◐	●	●	●	Particle-to-plant-to-profit-to-system chain, with explicit interrogation of how loss estimates are constructed

Coverage is scored as full (●), partial (◐) or absent (○).

## 2 Sources, fate, and soil-level processes: a condensed evidence base

This section compresses into a single narrative the three themes that occupied Sections 2 to 4 of the previous version, namely entry pathways, environmental fate, and soil-level consequences. The compression is deliberate and reflects the state of the field rather than the importance of the processes, because these questions have already been synthesized comprehensively and repeatedly, most recently in source-resolved and governance-oriented reviews that we do not attempt to duplicate (Kumar et al., 2020; Qi et al., 2020; Cai et al., 2023; Hoang et al., 2024; Roy et al., 2025; Boctor et al., 2025). Our selection criterion throughout is functional, in that we retain only the evidence that is subsequently load-bearing for the analysis of crop productivity, economic loss and supply-chain exposure, and we omit descriptive background that does not carry that weight. Three quantities matter downstream and are therefore reported here in detail: the magnitude and spatial heterogeneity of field contamination, which determines the exposure levels at which dose–response relationships must be evaluated; the direction of change in the particle population over time, which determines whether present-day measurements understate future exposure; and the soil-mediated mechanisms through which contamination reaches the plant, which determine whether yield penalties should be attributed to direct toxicity or to degraded soil function. The remaining material, including detailed accounts of polymer-specific fragmentation kinetics, aggregate-fraction partitioning and taxonomic shifts within the plastisphere, is signposted to the primary syntheses. Readers seeking a full treatment of sources and fate should consult those reviews, whereas readers concerned with productivity and its economic consequences will find the necessary foundations here. A second reason for compression is analytical rather than editorial, because the source-and-fate literature is expressed in particle counts per kilogram while the phytotoxicity literature is expressed in mass fractions, and the two cannot be reconciled without assumptions about particle size and density. Making that mismatch visible early is more useful than adding descriptive detail on either side of it. The tables in this section therefore preserve the quantitative anchors while the surrounding text has been substantially shortened. Where a contested claim exists, we retain the disagreement rather than the description. Everything that follows in Sections 3 to 7 depends on the numbers compiled here, and on very little else from the source literature.

### 2.1 Entry pathways and the magnitude of loading

Six pathways dominate microplastic delivery to farmland, and their relative importance is determined less by intrinsic emission strength than by local management intensity, which is why contamination maps track cropping systems rather than population density (Adhikari et al., 2024; Kumar et al., 2020; Roy et al., 2025). Plastic mulch films are the largest single source in intensively managed systems, because low-density polyethylene film raises soil temperature, conserves moisture and suppresses weeds, and because recovery of thin films after harvest is mechanically difficult and economically unrewarded (Qi et al., 2018; Huang et al., 2020). Long-term mulching leaves residues on the order of tens to hundreds of kilograms per hectare, and the most extreme documented consequence is in the cotton systems of Xinjiang, China, where mulched soils averaged approximately 12,589 particles per kilogram with more than ninety percent of particles below half a millimeter (Yu et al., 2023; Qi et al., 2020). Two qualifications matter for the later analysis. First, biodegradable films can generate microplastics faster than conventional polyethylene under field conditions, because they fragment readily while failing to mineralize *in situ*, so substitution is not automatically a reduction (Yang et al., 2022; Fan et al., 2022). Second, field-realistic accounting suggests that the proportional contribution of film has been overstated where macroplastic residue is conflated with the microplastic fraction actually generated within a given timeframe (Cai et al., 2023). Organic amendments constitute the second major pathway, and their significance lies in cumulative rather than instantaneous loading, as demonstrated by Chilean sludge-amended soils in which particle counts rose in proportion to the number of successive applications and by a twenty-three-year Australian biosolid trial reporting roughly twelve thousand particles per kilogram in the biosolid itself, several hundred per kilogram in amended soil, and far lower counts in unamended controls (Corradini et al., 2019; Adhikari et al., 2024). The polymer signature of amendment-derived particles is diagnostic, being enriched in polyester, polyamide and acrylic fibers shed during laundering, which permits source apportionment in mixed-contamination fields (Corradini et al., 2019). The remaining pathways are diffuse but not negligible, comprising irrigation, in which flood systems deposit and redistribute more particles than drip systems and drive smaller particles deeper into the profile; atmospheric deposition, which the same Australian work quantified as a continuous multi-year input; polymer-coated controlled-release fertilizers and seed coatings, which are an intentional primary source that fragments after nutrient release; and the weathering of greenhouse covers, shading nets and screens (Sattar et al., 2025; Xing et al., 2021; Adhikari et al., 2024; Bergmann et al., 2019; Bläsing and Amelung, 2018). A recent synthesis of 105 field datasets converted this source inventory into a practice-resolved risk matrix, concluding that direct inputs such as mulching pose the greatest long-term persistence risk while indirect inputs such as compost and biosolids carry the highest bioavailability risk, and identifying film thickness as a critical but under-regulated control point (Roy et al., 2025). **Table 3** compiles the representative magnitudes and polymer signatures that are used in the productivity and economic analyses below, and **Figure 1** depicts the converging pathways schematically.

**Table 3:** Principal entry pathways of microplastics into agricultural soils, representative reported magnitudes, and characteristic polymer signatures (studies published 2018–2026).

Pathway	Representative reported magnitude/abundance	Dominant polymers/morphology	Key sources
Plastic mulch films	Residues on the order of tens–hundreds of kg/ha; mulched soils up to ~12,589 particles/kg (Xinjiang cotton)	Polyethylene; films and fragments	Yu et al. (2023); Huang et al. (2020)
Sewage sludge/biosolids	~12,000 particles/kg in biosolids; several hundred/kg in amended soil vs low counts in controls (23-yr study)	Polyester, polyamide, acrylic fibers	Corradini et al. (2019); Adhikari et al. (2024)
Compost/municipal waste	Variable; elevated where feedstock contamination is uncontrolled	Mixed films and fragments	Bläsing and Amelung (2018); Sattar et al. (2025)
Irrigation water	Flood irrigation exceeds drip; promotes downward migration	Fibers and fragments; mixed	Garfansa et al. (2025); Xing et al. (2021)
Atmospheric deposition	Continuous input; measurable multi-year accumulation in amended soils	Fibers; small light fragments	Adhikari et al. (2024); Bergmann et al. (2019)
Coated fertilizers/seed coatings	Primary source; fragments after nutrient release	Polymer capsule shells	Bläsing and Amelung (2018); Kumar et al. (2020)
Practice-resolved risk ranking	Direct inputs (mulching) highest persistence risk; indirect inputs (compost, biosolids) highest bioavailability risk; film thickness identified as key regulatory lever	Practice-specific	Roy et al. (2025)

**Figure 1:**
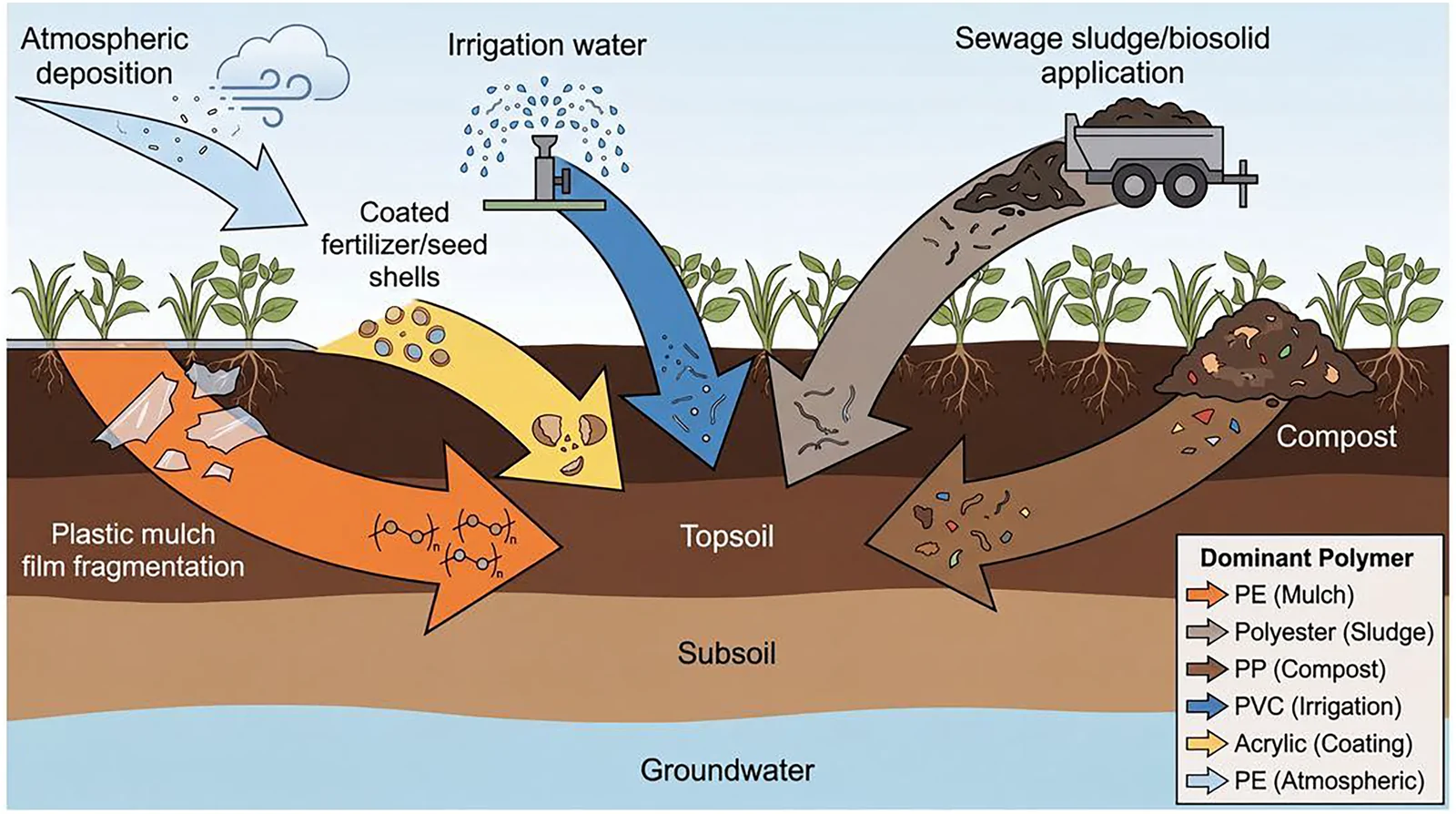
Conceptual schematic of microplastic entry pathways into agricultural soils. Depict a cross-sectional farmland scene with labelled arrows showing six converging inputs: plastic mulch film fragmentation, sewage sludge/biosolid application, compost, irrigation water, coated fertilizer/seed shells, and atmospheric deposition. The distinct colors or icons per pathway include a small legend indicating the dominant polymer associated with each input.

### 2.2 Weathering, transport, and the shifting particle population

Once deposited, particles are not static, and the direction of change is consistently toward smaller, more reactive and more mobile forms, which is the single most consequential fate observation for the risk analysis that follows. Freeze–thaw cycling, wetting and drying, tillage abrasion and mechanical stress progressively fracture larger fragments, so particle numbers increase over time even where new inputs cease entirely (Yang et al., 2022; Ya et al., 2021). Ultraviolet photo-oxidation and thermal oxidation cleave polymer chains and graft carbonyl and hydroxyl groups onto particle surfaces, raising specific surface area and the density of reactive sites, with the practical consequence that aged particles adsorb heavy metals and agrochemicals more strongly than pristine ones (Ren et al., 2021; Liu et al., 2024). Microbial colonization forms the plastisphere, a distinct habitat that slowly modifies polymer structure and that has itself become a focus of dedicated synthesis (Rillig and Lehmann, 2020; Rillig et al., 2024). Weathering also releases plasticizers, ultraviolet stabilizers and flame retardants into the surrounding soil at rates governed by temperature, pH and organic matter, so that the additive burden is a co-emitted rather than a separate hazard (Bläsing and Amelung, 2018; Kumar et al., 2020). Vertically, although topsoil retains the highest concentrations, particles migrate through preferential flow along cracks and root channels, in percolating water, and through the burrowing of soil fauna, with earthworms transporting particles into burrows and casts and dispersing them both downward and laterally (Rillig et al., 2017; O'Connor et al., 2019; Huerta Lwanga et al., 2016). Smaller particles migrate more readily, so weathering-driven size reduction actively promotes deeper transport and eventual contact with groundwater, and flood irrigation accelerates this relative to drip (Xing et al., 2021; Sattar et al., 2025). Spatially, national-scale survey data from China report mean farmland abundances of a few thousand particles per kilogram with very large dispersion among greenhouses, mulched fields and unmulched land, and this heterogeneity, not the mean, is what determines where productivity and economic losses will concentrate (Chen et al., 2022; Cai et al., 2023). Two implications carry forward. The first is temporal, in that the risk associated with a given historical plastic input intensifies rather than decays, so contemporary abundance measurements are a lagging indicator of eventual exposure. The second is metrological, in that field abundance is reported in particles per kilogram while phytotoxicity is tested in mass percentages, and converting between them requires assumptions about size distribution, shape and polymer density that are rarely stated, which is precisely the gap that undermines dose-calibrated economic estimation in Section 4. **Table 4** compiles the regional abundance data on which that discussion depends, and **Figure 2** illustrates the fate and transport processes across the profile.

**Table 4:** Reported abundance of microplastics in agricultural soils across selected regions and land uses (studies published 2019–2024).

Location/setting	Reported abundance (particles/kg)	Dominant type/size	Source
Xinjiang, China (mulched cotton)	Mean ~12,589 (range 4,198–47,420)	PE; >90% <0.5 mm	Yu et al. (2023)
National survey, China (farmland)	Mean ~2,462 ± 3,767 (± = 1 SD across sampled sites)	Films, fragments, fibers	Chen et al. (2022)
Nationwide review, China	Wide range across regions and depths	Films and fragments	Cai et al. (2023)
Chile (sludge-amended)	Rising with successive sludge applications	Fibers and films	Corradini et al. (2019)
Australia (biosolid-amended, 23 yr)	Several hundred/kg amended vs low controls	Fibers (polyester)	Adhikari et al. (2024)
Switzerland (floodplain soils)	Detected across floodplain profiles	Mixed polymers	Scheurer and Bigalke (2018)
Global (soil review)	Highly variable by region and method	Mixed polymers	Büks and Kaupenjohann (2020)

Values are reported as given in the cited sources. For the national Chinese survey, the ± term denotes one standard deviation of the site-level mean abundance across sampling locations, not a standard error or an analytical measurement uncertainty; the standard deviation exceeds the mean, indicating a strongly right-skewed distribution in which a minority of intensively managed sites accounts for a disproportionate share of the national burden (Chen et al., 2022). Abundances are not directly comparable across studies because extraction efficiency, minimum detectable particle size and identification method differ; the values should therefore be read as order-of-magnitude indicators rather than as a harmonized dataset.

**Figure 2:**
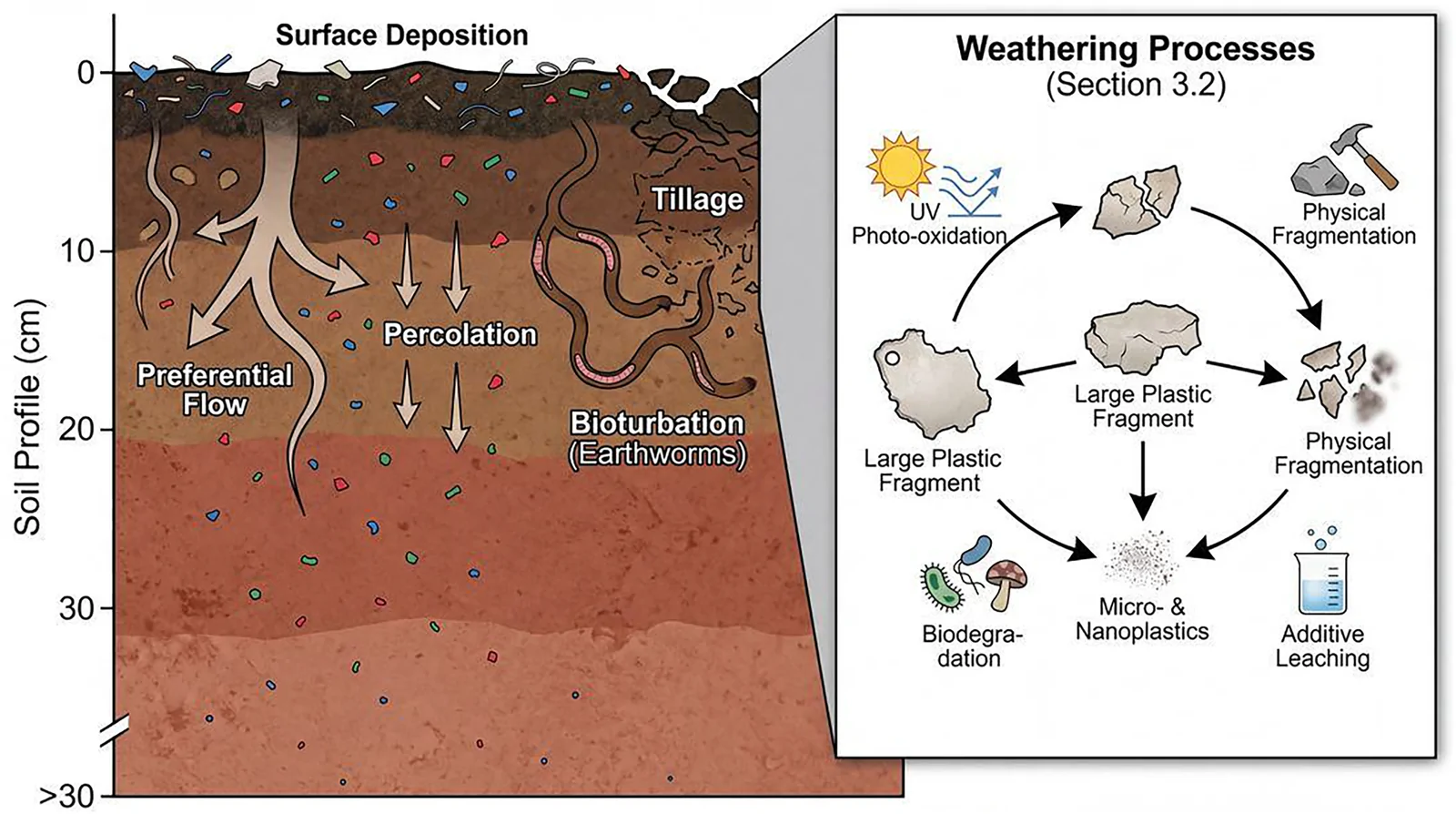
Fate, transformation, and vertical transport of microplastics across the soil profile. The image shows a soil column (0–30 cm and below) with surface deposition at top and a downward gradient of decreasing abundance, annotated with transport mechanisms (preferential flow, percolation, bioturbation by earthworms, tillage). A side panel illustrating weathering processes (UV photo-oxidation, physical fragmentation, biodegradation, additive leaching) converting large fragments into micro- and nanoplastics.

### 2.3 Soil-level consequences that mediate crop performance

Soil-level effects are treated here only insofar as they constitute the mechanistic bridge between contamination and yield, because it is this mediation, rather than direct particle toxicity alone, that determines the magnitude of productivity loss under field conditions. Physically, plastic particles differ from mineral grains in density, shape and surface properties, so their incorporation alters bulk density, porosity, aggregate stability and water-holding capacity, with fibers creating channels that increase evaporation and films forming barriers that impede infiltration and gas exchange (de Souza Machado et al., 2018, 2019; Wan et al., 2019; Lozano and Rillig, 2020). Critically for interpretation, the sign of these effects is polymer- and dose-dependent, since foundational experimental work showed that different polymer types increased or decreased water-stable aggregation, which means that a single average effect size conceals genuinely opposing responses (de Souza Machado et al., 2019). Biologically, plastic surfaces recruit a distinct microbial community whose composition diverges from the surrounding bulk soil, and the functional consequences include altered enzyme activity, with suppression of urease, phosphatase and dehydrogenase reported in several studies alongside enzyme-specific exceptions (Rillig and Lehmann, 2020; Fei et al., 2020; Awet et al., 2018; Rillig et al., 2024). The nitrogen cycle is disturbed in a manner directly relevant to yield, because interference with nitrifying and denitrifying communities and increased ammonia volatilization reduce the nitrogen actually available for grain filling, providing a nutritional rather than toxicological route to productivity loss (Sarfraz et al., 2025). Carbon cycling responds in both directions depending on polymer type and dose, and comparative work confirms that biodegradable particles are not functionally benign relative to conventional ones (Ma et al., 2023; Fan et al., 2022). Soil fauna occupy a dual position as both victims and vectors, since earthworms suffer reduced growth and impaired reproduction under exposure while simultaneously redistributing particles through burrowing and casting, and field evidence has documented transfer of plastic debris along a terrestrial food chain beginning with soil detritivores (Huerta Lwanga et al., 2016, 2017; Boots et al., 2019). Other fauna are affected in ways with functional consequences, including impeded movement of springtails through soil pore networks and documented sensitivity of protists (Kim and An, 2019; Rillig and Bonkowski, 2018). The analytically important point is that these soil-level disturbances are not additive with direct phytotoxicity but partly redundant with it, since a study that measures biomass reduction in contaminated soil captures the sum of direct injury and degraded soil function without separating them. This confounding is one reason why laboratory effect sizes cannot be transferred to field yield predictions without adjustment, an issue taken up in Sections 3.5 and 4.1. **Figure 3** integrates these soil-level and organismal effects with the plant-level mechanisms described next.

**Figure 3:**
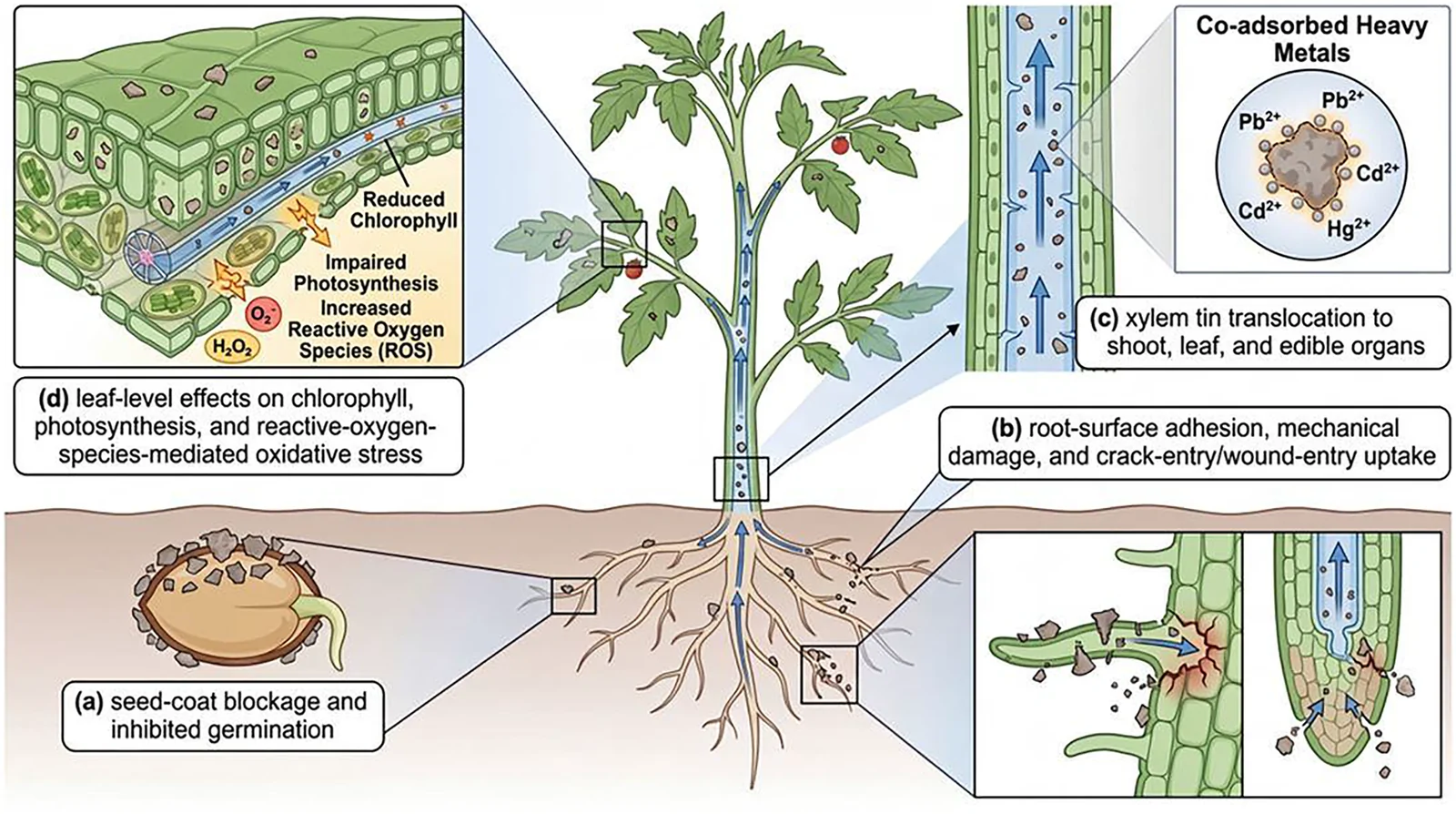
Integrated mechanisms of microplastic phytotoxicity and uptake in crops. A plant with: **(a)** seed-coat blockage and inhibited germination; **(b)** root-surface adhesion, mechanical damage, and crack-entry/wound-entry uptake; **(c)** xylem translocation to shoot, leaf, and edible organs; **(d)** leaf-level effects on chlorophyll, photosynthesis, and reactive-oxygen-species-mediated oxidative stress. It also includes a small inset showing co-adsorbed heavy metals on a particle surface.

## 3 Phytotoxicity and the erosion of crop productivity

### 3.1 Seed germination, root architecture, and early establishment

The earliest stages of the plant life cycle are especially vulnerable to microplastics, and disturbances at germination and establishment propagate into lifelong deficits in growth and yield. Microplastics can adhere to seed surfaces and physically block the seed-coat pores through which water and oxygen are imbibed, thereby delaying or reducing germination in a range of species, as first clearly demonstrated for cress seedlings (Bosker et al., 2019). Once seedlings emerge, particles accumulate on root surfaces and within the rhizosphere, where they can mechanically damage root tissue, obstruct root hairs, and impede the uptake of water and nutrients (Wang et al., 2022; Qi et al., 2018). Reported responses include inhibited root elongation, altered root architecture, and reduced root biomass, with the severity generally increasing at higher concentrations and smaller particle sizes (Wang et al., 2022; de Souza Machado et al., 2019). Experiments on common bean documented growth responses to microplastic-contaminated soil, illustrating that legumes as well as cereals are susceptible at the establishment stage (Meng et al., 2021). Nanoplastics and smaller microplastics tend to be more damaging than larger particles because their greater relative surface area intensifies contact and reactivity at the root interface (Wang et al., 2022). Importantly, responses are not universally negative, since some studies report negligible or even mildly stimulatory effects at low doses, reflecting hormesis and the strong dependence on polymer, dose, and species (Fan et al., 2022; Ng et al., 2018). Wheat exposed to plastic mulch film residues showed measurable growth penalties in both vegetative and reproductive development, linking a common agricultural input directly to crop performance (Qi et al., 2018). The concentration-dependence and species-specificity of germination and root effects are recurring and important qualifications (Cai et al., 2023). Even so, the balance of evidence indicates that early-stage exposure frequently compromises establishment. Deficits incurred at this stage set an upper bound on subsequent productivity. Quantifying how large these establishment penalties are in aggregate has recently become possible, because a global meta-analysis of 63 publications and 3,268 observations found that crop morphological traits were significantly inhibited overall, with root morphology reduced by approximately 6.65 percent, shoot morphology by approximately 9.7 percent and fruit traits by approximately 13.34 percent (Sun H. et al., 2024). The ordering of these effect sizes is itself informative for the productivity argument developed in this review, because reproductive and harvestable organs were suppressed more strongly than vegetative or below-ground organs, implying that studies terminating at the seedling stage systematically understate the yield-relevant consequence of early exposure. It also indicates that the establishment deficits described above are not merely transient setbacks from which plants fully recover, since the penalty appears to widen rather than narrow as the crop progresses toward harvest.

### 3.2 Photosynthesis, nutrient acquisition, and biomass penalties

As plants develop, microplastics continue to constrain productivity by impairing photosynthesis, disrupting nutrient acquisition, and imposing metabolic costs that manifest as reduced biomass and yield. Exposure has been shown to lower chlorophyll content and photosynthetic efficiency, both through direct effects on leaf physiology and through indirect effects mediated by root damage and nutrient limitation (Garfansa et al., 2025; Zhu et al., 2025). The landmark global meta-analysis by Zhu and colleagues synthesized more than three thousand data points and estimated that microplastic exposure reduces photosynthesis by roughly seven to twelve percent across terrestrial plants, marine algae, and freshwater algae (Zhu et al., 2025). Translating this suppression into agronomic terms, the same study projected annual losses of staple crop production on the order of one hundred to three hundred and sixty million tons, a magnitude comparable to major climatic stressors (Zhu et al., 2025). At the physiological level, microplastics induce the accumulation of reactive oxygen species, triggering oxidative stress that damages membranes through lipid peroxidation and impairs enzymatic function (Wang et al., 2022). Plants respond by upregulating antioxidant enzymes and stress-related pathways, diverting resources away from growth and reproduction toward defence (Wang et al., 2022; Fan et al., 2022). Microplastics also interfere with nutrient acquisition by altering rhizosphere chemistry and root function, reducing the uptake of nitrogen, phosphorus, and micronutrients essential for grain filling (Sarfraz et al., 2025; Fei et al., 2020). Controlled experiments have documented dose-dependent reductions in chlorophyll and biomass across multiple crop species exposed to elevated particle concentrations (Qi et al., 2018; Wang et al., 2022). The convergence of oxidative, nutritional, and photosynthetic stress explains why biomass penalties are so consistently observed across crops such as lettuce, wheat, maize, and rice (Zhu et al., 2025; Meng et al., 2021). **Table 3** compiles representative phytotoxic effects reported for major crops. These productivity penalties are the biophysical origin of the economic losses examined subsequently. Two independent quantitative syntheses can now be compared directly on this endpoint, which is analytically valuable because they were assembled from different observation pools and by different methods. The photosynthesis-focused synthesis reported suppression of roughly 7.05 to 12.12 percent across terrestrial plants and algae, whereas the trait-level agricultural meta-analysis reported photosynthetic inhibition of approximately 9.1 percent alongside reductions in respiration of approximately 34.44 percent and transpiration of approximately 14.74 percent in crop species specifically (Zhu et al., 2025; Sun H. et al., 2024). The convergence of the two photosynthetic estimates within a narrow band strengthens confidence in the central effect size, while the divergence between photosynthetic and respiratory responses indicates that the carbon balance, rather than photosynthetic capacity alone, is what is disturbed. This distinction matters for the economic translation attempted in Section 4, because a plant that fixes less carbon and simultaneously respires substantially more will exhibit a biomass penalty larger than photosynthetic suppression alone would predict, whereas a plant that merely delays growth may still reach a comparable final yield.

### 3.3 Uptake, translocation, and accumulation in edible tissues

A particularly consequential dimension of phytotoxicity is the capacity of small plastic particles to enter plant tissues and translocate to edible organs, bridging agronomic and food-safety concerns. It was long assumed that plant cell walls formed an impenetrable barrier, but accumulating evidence demonstrates that nanoplastics and submicron microplastics can be internalized by roots and transported upward through the vascular system (Wang et al., 2022; Yin et al., 2026). Entry is thought to occur through crack-entry points at sites of lateral root emergence, through apoplastic and symplastic pathways across the root epidermis, and, for the smallest particles, through cellular uptake mechanisms (Wang et al., 2022). Once inside the root, particles can move via the xylem to stems, leaves, and in some cases fruits and grains, with translocation shown to be dose-dependent and size-selective, favoring smaller particles (Wang et al., 2022). A recent controlled study revealed that root wounds dramatically facilitate uptake, with deep injuries providing rapid pathways for polystyrene particles to accumulate in taro corms and maize stems at substantial concentrations, underscoring the relevance of farming practices that damage roots (Yin et al., 2026). This finding is agronomically significant because transplanting, cultivation, and pest activity routinely wound roots under field conditions (Yin et al., 2026). The internalization of particles carries a dual risk, simultaneously harming the plant and introducing plastics into the human food chain through consumption of contaminated produce (Huerta Lwanga et al., 2017; Wang et al., 2022). Uptake also facilitates the co-transport of adsorbed contaminants such as heavy metals into edible tissues, compounding the hazard (Liu et al., 2024). The quantitative extent of translocation into staple grains under realistic field conditions nonetheless remains uncertain and is an active area of investigation (Cai et al., 2023). This uncertainty is a key knowledge gap addressed later in the review. The phenomenon of uptake fundamentally links soil contamination to dietary exposure.

### 3.4 Amplified injury under co-contamination

Microplastics seldom act alone in agricultural soils, and their interaction with co-occurring contaminants frequently amplifies phytotoxicity beyond the sum of individual effects. Owing to their large specific surface area, hydrophobic domains, and charged functional groups, microplastics readily adsorb heavy metals, pesticides, and organic pollutants, functioning as vectors that concentrate and redistribute these toxicants (Liu et al., 2024; Ren et al., 2021). Aging enhances this vector effect, because weathered particles present more oxygen-containing groups that increase metal-binding affinity, so environmental exposure tends to strengthen rather than weaken the interaction over time (Liu et al., 2024; Ren et al., 2021). Studies on the co-exposure of microplastics and metals have shown that outcomes are concentration-dependent, with adsorption sometimes reducing bioavailability at low metal concentrations yet producing intensified combined toxicity at higher concentrations (Liu et al., 2024). The adsorption affinity of microplastics differs among metals and depends strongly on polymer type, particle size, and degree of aging, which makes combined-pollution outcomes difficult to predict from single-contaminant data (Liu et al., 2024). The combined presence of microplastics and agrochemicals similarly amplifies ecological stress, altering the fate, mobility, and bioavailability of pesticides and increasing the risk of bioaccumulation (Liu et al., 2024; Yang et al., 2022). These interactions matter agronomically because they can convert sub-toxic concentrations of individual contaminants into a jointly injurious mixture (Liu et al., 2024). They also complicate risk assessment, which has traditionally evaluated contaminants in isolation (Ng et al., 2018). The recognition that microplastics act as mobile platforms for other pollutants is among the more policy-relevant insights of recent research (Liu et al., 2024). It reframes microplastics as both direct stressors and enablers of secondary harm. This compounding role is revisited in the discussion of food safety. Field-scale confirmation of these interactions remains an important research need. The magnitude of this amplification has now been estimated systematically rather than inferred from individual pairings, since a global meta-analysis combined with machine-learning analysis of 3,346 biological endpoints drawn from 68 studies concluded that co-exposure to microplastics and other soil pollutants significantly exacerbates toxicity to crops relative to either stressor alone (Wang et al., 2024). This finding is directly consequential for the economic analysis that follows, because it means that yield-loss coefficients derived from single-contaminant experiments will understate real-world penalties in the very soils where losses are most likely to be economically significant, namely intensively managed, long-cultivated land carrying legacy burdens of metals, pesticide residues and veterinary antibiotics. It also implies that the marginal benefit of microplastic mitigation is greater in co-contaminated systems than in clean ones, which is a useful criterion for prioritizing intervention where budgets are constrained.

### 3.5 Dose–response, particle-size dependence, and the field-realism discount

The preceding subsections establish that microplastics injure crops, but the economic analysis in Section 4 requires something stronger, namely a defensible relationship between an exposure level that actually occurs in farmland and a proportional loss of harvestable yield, and it is worth stating plainly that no such relationship has yet been established with confidence. The obstacle begins with units, because field surveys report abundance as particles per kilogram of dry soil while controlled experiments almost universally dose in mass percentages, and conversion between the two requires assumptions about the size distribution, morphology and polymer density of the particle population that are seldom measured and almost never reported (Chen et al., 2022; Sun H. et al., 2024). A soil containing several thousand small polyethylene fragments per kilogram may correspond to a mass fraction several orders of magnitude below the one to ten percent w/w doses that dominate the experimental literature, which means that much of the mechanistic evidence base characterizes an exposure regime that few fields experience. The consequence is not that laboratory findings are invalid but that the shape of the dose–response curve in the environmentally relevant range remains largely unobserved, and the widely reported non-monotonic and occasionally hormetic responses at low doses make linear extrapolation downward from high-dose experiments unsafe (Fan et al., 2022; Ng et al., 2018). Particle size compounds the problem in the opposite direction, because smaller particles are consistently more damaging per unit mass owing to greater specific surface area and greater capacity for internalization, and because weathering continuously shifts the field population toward smaller sizes, so a mass-based exposure metric held constant over time nonetheless corresponds to rising toxicity (Wang et al., 2022; Ya et al., 2021). Exposure duration introduces a third asymmetry, since most experiments run for a single season or less while field contamination accumulates over decades, and the twenty-three-year Australian biosolid record demonstrates that the relevant timescale for accumulation is measured in decades rather than seasons (Adhikari et al., 2024). A fourth consideration is that experimental particles are typically pristine spheres of a single polymer, whereas field particles are aged, irregular, chemically heterogeneous and carrying adsorbed co-contaminants, and the co-exposure evidence indicates that this difference biases laboratory results downward rather than upward (Liu et al., 2024; Wang et al., 2024). These four adjustments do not point in the same direction, which is precisely why a simple correction factor cannot be applied: dose overestimation inflates laboratory effect sizes, whereas particle pristineness, short duration and single-contaminant design deflate them. We therefore adopt the position throughout Section 4 that published global loss estimates are best interpreted as scoping calculations whose central values are plausible but whose confidence intervals are unquantified, and we specify in Section 8 the experimental programme that would be required to replace them with calibrated coefficients. **Table 5** accordingly reports documented effects together with the exposure basis on which each was obtained, so that readers can see which entries rest on field-realistic conditions. Recognizing this limitation is not a retreat from the argument of this review but a precondition for making it credible.

**Table 5:** Representative documented phytotoxic effects of microplastics and nanoplastics on major crops (selected studies, 2018–2026), with the exposure basis on which each observation was obtained.

Crop/plant	Polymer & particle type	Principal reported effect	Exposure basis	Source
Wheat (Triticum aestivum)	Plastic mulch film residues/MPs	Reduced vegetative and reproductive growth	Pot study, film residues at field-plausible loading	Qi et al. (2018)
Maize (Zea mays)	PS microplastics	Uptake into stems via root wounds	Controlled hydroponic/soil exposure	Yin et al. (2026)
Taro (Colocasia esculenta)	PS (1–5 µm)	Accumulation in edible corms via deep root wounds	Controlled exposure with imposed root wounding	Yin et al. (2026)
Common bean (Phaseolus vulgaris)	Mixed microplastics	Altered growth in contaminated soil	Pot study, spiked soil	Meng et al. (2021)
Cress (Lepidium sativum)	Microplastic particles	Delayed germination; reduced root growth	Germination assay, suspension exposure	Bosker et al. (2019)
Multiple crops (meta-analysis)	Various polymers	~7–12% reduction in photosynthesis	Meta-analysis of >3,000 data points, mixed doses	Zhu et al. (2025)
Multiple crops (meta-analysis)	Various polymers	Fruit traits −13.34%; shoot −9.7%; root −6.65%; photosynthesis −9.1%; respiration −34.44%	63 publications; 3,268 observations; predominantly spiked-soil experiments	Sun H. et al. (2024)
Multiple crops (meta + machine learning)	MPs with co-occurring soil pollutants	Significant exacerbation of toxicity under co-exposure relative to single stressors	3,346 endpoints from 68 studies	Wang et al. (2024)
Leafy greens/general	PE, PS micro- and nanoplastics	Biomass reduction; tissue uptake	Mixed laboratory and pot studies	Wang et al. (2022)

## 4 From yield penalties to economic losses

This section carries the analytical weight of the review, and it has been substantially expanded because the translation of biological injury into monetary loss is where the literature is thinnest and where the contribution of an integrative synthesis is greatest. We proceed in five steps. Section 4.1 dissects the inferential chain by which published global loss figures are constructed and identifies the assumptions that govern their reliability. Section 4.2 examines the production and revenue estimates themselves and the price-transmission mechanics that separate a production shortfall from a revenue loss. Section 4.3 turns from the top-down global perspective to bottom-up farm accounts, using published cost–benefit analyses of mulch film management to show why contamination persists despite being collectively costly. Section 4.4 addresses the hidden costs that fall outside conventional yield accounting, and Section 4.5 asks who actually bears them. Section 4.6 consolidates the discussion into a structured accounting framework that specifies, for each cost category, the appropriate valuation method and the current state of evidence. Throughout, we distinguish carefully between quantities that have been measured, quantities that have been modelled, and quantities that have merely been asserted, because conflating the three is the principal weakness of the existing economic literature on agricultural plastics.

### 4.1 How global loss estimates are constructed, and where their uncertainty resides

The headline figure that microplastic pollution may cost the world between roughly 110 and 361 million tons of staple crop production annually is now widely cited, and it deserves close examination rather than repetition, because the number is the product of a five-step inferential chain in which each step introduces an assumption that is defensible individually but compounding in aggregate (Zhu et al., 2025). The first step estimates a mean effect of microplastic exposure on photosynthesis by pooling more than three thousand observations from controlled studies, yielding a central suppression of roughly 7 to 12 percent, and this step is the most robust of the five because it rests on a large and internally consistent observation pool. The second step maps photosynthetic suppression onto biomass accumulation, and here the assumption of proportionality is the weakest link, since photosynthetic rate measured instantaneously on a single leaf does not translate linearly into whole-plant carbon gain when canopy architecture, sink limitation, compensatory growth and phenological plasticity intervene; a plant whose instantaneous assimilation is depressed may partially recover through extended duration of growth, whereas one whose respiration is simultaneously elevated by roughly a third will lose more carbon than the assimilation figure implies (Sun H. et al., 2024). The third step converts biomass into harvestable yield through an implicit harvest-index assumption, and the trait-level meta-analysis provides indirect evidence that this assumption is conservative rather than generous, because reproductive and fruit traits were inhibited more strongly than vegetative traits, suggesting that harvest index may itself decline under exposure rather than remaining constant (Sun H. et al., 2024). The fourth step scales the per-plant penalty to global production by applying it across planted area and current output, which requires an exposure surface, and since spatially resolved soil microplastic concentrations exist for only a small number of countries, this step necessarily substitutes modelled or interpolated exposure for measured exposure across most of the world. The fifth step monetizes the physical shortfall at prevailing commodity prices, an operation discussed in Section 4.2 that is considerably less straightforward than it appears. Set against this chain are the four field-realism adjustments developed in Section 3.5, which do not resolve into a single directional bias: high experimental doses inflate the effect size, while pristine particles, short exposure durations and single-contaminant designs deflate it. The honest conclusion is that the direction of the net bias in current global estimates is unknown, and that the published range should therefore be read as an order-of-magnitude scoping exercise establishing that the problem is macroeconomically non-trivial, rather than as a calibrated forecast suitable for cost–benefit arithmetic. It is worth noting that independent commentary on the global photosynthesis estimate has raised precisely this concern about the chain of extrapolations, and the appropriate response is not to discard the figure but to state its epistemic status explicitly whenever it is used. We adopt that practice for the remainder of this section, and Section 8 sets out the specific measurements that would convert scoping estimates into decision-grade ones.

### 4.2 Quantifying production and revenue losses

The translation of biophysical yield penalties into monetary losses is the dimension of microplastic contamination most directly relevant to farmers, markets, and policymakers, yet it has historically received the least rigorous quantification. The most influential recent estimate derives from the global photosynthesis meta-analysis, which projected that microplastic-induced suppression could reduce annual staple crop production by approximately one hundred to three hundred and sixty million tons, a loss whose scale rivals that attributed to climate variability in recent decades (Zhu et al., 2025). Interpreted through prevailing commodity prices, production shortfalls of this order imply revenue losses running into billions of dollars annually across the wheat, rice, and maize sectors (Zhu et al., 2025). A dedicated economic assessment estimated production and revenue losses for rice, wheat, maize, tomato, and peas attributable to microplastics over a multi-year period, providing one of the few crop-specific monetized analyses available (Sutanto et al., 2024). Such estimates remain sensitive to assumptions about dose-response relationships, baseline contamination, and the counterfactual of a plastic-free system, so they should be read as indicative rather than definitive (Sutanto et al., 2024; Cai et al., 2023). The modelling nonetheless consistently indicates that even modest percentage yield declines, when applied across the vast area planted to staple crops, aggregate into economically material losses (Zhu et al., 2025). Importantly, the same analyses suggest that partial mitigation could recover a meaningful share of these losses, with a modest reduction in environmental microplastic levels projected to avert tens of millions of tons of lost production (Zhu et al., 2025). This positive framing reframes mitigation expenditure as an investment with quantifiable returns rather than a pure cost (Gontard, 2025). **Table 4** collates representative yield and economic-loss estimates from the recent literature. These figures establish that microplastic contamination has moved from an ecological abstraction to a measurable drag on agricultural output. The economic case for intervention rests substantially on this evidence. It also motivates the more rigorous economic modelling identified later as a research priority. Three mechanisms complicate the conversion of a physical shortfall into a revenue loss, and none is addressed in the current literature. The first is price transmission, because staple crop demand is price-inelastic, so a production shortfall raises the market price and partially offsets the revenue effect for producers who still have output to sell, with the result that aggregate producer revenue may fall by considerably less than production, or in extreme cases rise, while consumers and net-importing countries absorb the welfare loss instead; a loss estimate that multiplies tons foregone by an unchanged price therefore measures neither producer income loss nor social welfare loss but a hybrid of the two. The second is the distribution of the shortfall, since contamination is spatially concentrated rather than uniform, and a loss borne disproportionately by growers in a small number of intensively mulched regions has entirely different distributional and market consequences from the same tonnage spread thinly across all production. The third is attribution, because microplastic-induced losses are estimated against a counterfactual of a plastic-free system in which mulching would also be absent, and since mulching itself raises yields substantially in arid and cold regions, the correct counterfactual is not current yield plus the loss but the yield that would have been obtained without plasticulture at all, a comparison that materially reduces the net penalty attributable to plastic use and that we develop quantitatively in Section 4.3. Recognizing these three mechanisms does not weaken the case for concern, but it does relocate it, because the strongest economic argument against contamination is not the annual flow of foregone revenue but the progressive and largely irreversible depreciation of soil productive capital, which conventional annual accounting does not capture at all.

### 4.3 Farm-level cost structures and the plasticulture trade-off

If contamination imposes real costs, the persistence and continued expansion of the practices that generate it requires explanation, and the explanation is found in the structure of farm-level accounts rather than in ignorance or irrationality on the part of growers. Meta-analytic evidence from northwestern China shows that plastic film mulching increased economic return by an average of 29.5 percent relative to unmulched cultivation, with the benefit concentrated in spring maize at approximately 71.1 percent and effectively absent for spring wheat, which establishes both the magnitude of the private incentive and its crop specificity (Ma et al., 2018). A recent cost–benefit analysis of mulch film management in northern China quantifies the resulting trade-off with unusual precision, reporting a net benefit of CNY 2,955.0 per hectare, equivalent to approximately USD 413.7 per hectare, for conventional film relative to no mulching (Hao et al., 2024). The same analysis evaluated the two principal environmental interventions currently promoted in China and reached a conclusion that should reframe mitigation policy: application of thicker, more recoverable film yielded a net benefit of CNY 3,208.8 per hectare, approximately USD 449.2 per hectare, which exceeds conventional film by CNY 253.8 per hectare, whereas substitution with biodegradable film yielded only CNY 2,244.6 per hectare, approximately USD 314.2 per hectare, and is therefore unprofitable relative to the conventional benchmark and dependent on subsidy for adoption (Hao et al., 2024). The most analytically revealing figure in that study, however, concerns the composition rather than the level of benefits, because yield increase accounted for approximately 96.0 percent of the total benefit of the thicker-film option and 99.7 percent of the biodegradable option, while external benefits comprising avoided plastic pollution and the value of recycling and substitution amounted to roughly 0.9 percent (Hao et al., 2024). That single proportion explains the entire policy problem addressed by this review, because it demonstrates that within the accounting frame that actually governs farm and subsidy decisions, the contamination externality is not merely undervalued but effectively invisible, contributing under one percent of measured benefit while the productivity gain that drives adoption contributes almost all of it. Evidence from a very different production context reinforces the same structural conclusion, since a partial-budget and sensitivity analysis of biodegradable mulch adoption in Tennessee pumpkin production found that profitability hinged on the price of the biodegradable film, labor costs, and price discounts arising from mulch adhesion to fruit, with transition to biodegradable film improving profits under current prices and no discount but reducing them once a five percent price discount was assumed (Velandia et al., 2020a). Survey work in the same region found that grower willingness to pay for biodegradable mulch lies below prevailing market prices, which identifies a persistent adoption gap that information campaigns alone will not close (Velandia et al., 2020b). Read together, these three literatures deliver a coherent and policy-relevant message: the cheapest available mitigation is the one that is already privately profitable, namely thicker and mechanically recoverable film supported by functioning collection infrastructure, while material substitution is currently a subsidy-dependent option whose contamination benefit is in any case contested by the evidence that biodegradable films can fragment faster than conventional ones (Fan et al., 2022; Yang et al., 2022). **Table 6** incorporates these farm-level figures alongside the global estimates so that the two scales of analysis can be read against each other.

**Table 6:** Representative estimates of yield and economic losses and of farm-level returns to plasticulture management associated with microplastic contamination (2018–2025).

Metric/scope	Reported estimate	Basis	Source
Global photosynthesis reduction (all ecosystems)	~7.05–12.12% reduction	Meta-analysis of >3,000 observations	Zhu et al. (2025)
Global staple crop production loss (annual)	~109.73–360.87 million tons	Modelled extrapolation from photosynthetic effect size	Zhu et al. (2025)
Wheat, rice, maize yield loss (modelled)	Several percent, scenario-dependent	Modelled	Zhu et al. (2025)
Recoverable loss under ~13% MP reduction	Tens of Mt of crop production averted	Modelled scenario	Zhu et al. (2025)
Crop-specific revenue loss (rice, wheat, maize, tomato, peas)	Monetized multi-year losses estimated	Commodity-level economic assessment	Sutanto et al. (2024)
Crop trait responses (fruit, shoot, root, photosynthesis)	−13.34%, −9.7%, −6.65%, −9.1% respectively	Meta-analysis, 63 publications, 3,268 observations	Sun H. et al. (2024)
Economic return to plastic film mulching	+29.5% on average; +71.1% spring maize; no gain spring wheat	Meta-analysis of field trials, NW China	Ma et al. (2018)
Net benefit, conventional mulch film	CNY 2,955.0/ha (~USD 413.7/ha)	Cost–benefit analysis, field survey data, N China	Hao et al. (2024)
Net benefit, thicker (recoverable) film	CNY 3,208.8/ha (~USD 449.2/ha); +CNY 253.8/ha vs conventional	Cost–benefit analysis	Hao et al. (2024)
Net benefit, biodegradable film substitution	CNY 2,244.6/ha (~USD 314.2/ha); unprofitable vs conventional	Cost–benefit analysis	Hao et al. (2024)
Share of accounted benefit attributable to external/environmental gains	~0.9% (vs ~96–99.7% from yield increase)	Cost–benefit decomposition	Hao et al. (2024)
Profitability of biodegradable mulch adoption	Positive at current prices without discount; negative under a 5% price discount	Partial budget and sensitivity analysis, US pumpkin	Velandia et al. (2020a)
Framing of plastics as an economic burden on agri-food	Long-term particle footprint costs	Conceptual/perspective	Gontard (2025)

### 4.4 Hidden costs: remediation, quality downgrading, and market access

Beyond the visible loss of yield lies a set of hidden costs that broaden the economic footprint of microplastic contamination well past the farm gate. Remediation and soil-restoration measures, including the application of biochar, the recovery of residual films, and the transition to alternative materials, impose direct expenditures on producers that may not be recouped through higher yields in the short term (Wu et al., 2024; Gontard, 2025). Contamination can also downgrade produce quality, reducing the market value of crops through diminished size, altered appearance, or the presence of plastic particles in edible tissue (Sutanto et al., 2024; Yin et al., 2026). Where contamination becomes detectable or exceeds emerging standards, producers may face restricted market access, particularly in export markets governed by increasingly stringent food-safety and sustainability requirements (Gontard, 2025). The certification and monitoring needed to demonstrate compliance add transaction costs that can fall disproportionately on smallholders with limited capacity to absorb them (Gontard, 2025; FAO, 2025). There are further downstream costs associated with contamination of the food chain, since particles that enter crops and livestock ultimately reach consumers and raise food-safety concerns that carry economic weight (Huerta Lwanga et al., 2017; Sutanto et al., 2024). Framing plastic pollution as an economic as well as an ecological problem has become increasingly common, with the agri-food sector identified as both a major contributor to and a bearer of these costs (Gontard, 2025). These distributed and often externalized costs are rarely captured in farm-level accounting, which causes the true economic burden to be systematically underestimated (Gontard, 2025). A full plastic-particle footprint accounting, as recently proposed, would render these hidden costs visible and support better decision-making (Gontard, 2025). Recognizing the breadth of these costs is essential to designing proportionate policy. The economic dimension thus extends from the individual field to the structure of markets themselves. These categories can be made more tractable by distinguishing flow costs from stock costs, a distinction absent from the current literature but essential to proportionate policy. Flow costs recur annually and include yield-related revenue reduction, remediation expenditure, quality downgrading and the transaction costs of certification and monitoring, and they are in principle observable in farm accounts, even though they are seldom attributed to contamination because no grower currently measures soil microplastic burden. Stock costs, by contrast, represent the depreciation of soil natural capital, since particles accumulate over decades, fragment progressively into more reactive forms, and cannot be economically removed at field scale with any currently validated technology, which means that each season of continued loading transfers an irreversible liability to future production rather than imposing a recoverable annual charge. The twenty-three-year biosolid record demonstrates that this stock accumulates measurably over an ordinary management lifetime, and the weathering evidence indicates that its toxicological potency increases as it ages, so the appropriate financial analogue is a depreciating asset with a rising maintenance burden rather than a recurring operating expense (Adhikari et al., 2024; Ren et al., 2021). This reframing has a practical implication for appraisal, because discounting a stream of small annual flow costs will always understate a problem whose principal economic characteristic is the progressive and irreversible impairment of the asset on which all future flows depend.

### 4.5 Distributional incidence: who actually bears the loss

Aggregate loss figures obscure the question that determines political feasibility, namely who pays, and the available evidence suggests that the incidence of microplastic costs is markedly regressive across at least four dimensions. The first is spatial, because contamination concentrates in intensively mulched systems and the most severe documented burdens occur in regions such as Xinjiang where film use is not discretionary but agronomically necessary for cotton production under arid conditions, so the growers facing the highest exposure are precisely those with the fewest viable alternatives (Yu et al., 2023; Sattar et al., 2025). The second is scale-related, since compliance with emerging standards requires sampling, laboratory analysis and certification whose costs are largely fixed rather than proportional to area, and fixed compliance costs fall disproportionately on smallholders, who dominate production in most of the regions where the evidence base is weakest (FAO, 2025; Gontard, 2025). The third dimension operates through markets rather than fields, because price transmission means that a contamination-driven production shortfall is partly borne by consumers and by net food-importing countries that never used the plastic in question, converting a local management externality into an international welfare transfer whose magnitude has never been estimated. The fourth is temporal and intergenerational, since the stock character of contamination described in Section 4.4 means that the beneficiaries of current plasticulture and the bearers of its eventual productivity cost are not the same cohort of farmers, which weakens the private incentive for voluntary abatement in exactly the way that standard analyses of soil degradation predict. These four channels interact in ways that matter for instrument design. A uniform levy on agricultural film would be regressive in the second and first dimensions simultaneously, whereas a subsidy directed at thicker recoverable film and collection infrastructure exploits the finding that this option is already privately profitable and therefore requires only a modest transfer to shift behavior (Hao et al., 2024). Conversely, subsidizing biodegradable substitution transfers public funds toward an option that is currently unprofitable, whose contamination benefit is contested, and whose adoption gap reflects a genuine willingness-to-pay shortfall rather than a market failure (Velandia et al., 2020b; Fan et al., 2022). The distributional analysis therefore converges with the cost-effectiveness analysis on the same policy ordering, which is a reassuring result and one that could not have been reached from the global loss estimates alone. It also identifies the most urgent measurement gap, since none of these incidence channels can be quantified without spatially resolved exposure data of a kind that currently exists for only a handful of countries.

### 4.6 A structured accounting framework for microplastic costs in agriculture

The preceding subsections can be consolidated into an explicit accounting framework, and doing so serves two purposes: it makes visible which cost categories are currently quantified and which are merely asserted, and it specifies the valuation method appropriate to each, so that future studies can be compared rather than merely accumulated. **Table 7** sets out this framework across six cost categories, distinguishing for each the level at which the cost arises, the valuation method that is methodologically appropriate, the current state of empirical evidence, and the principal source of uncertainty. Several features of the resulting picture are worth stating directly. Only two of the six categories, namely yield-related revenue loss and the direct cost of film management, currently rest on quantitative evidence, and of these only film management rests on measured rather than modelled data. Three categories, namely quality downgrading, market access restriction and compliance cost, are widely asserted in the literature but supported by no published quantification for microplastics specifically, and until such quantification exists they should be described as plausible mechanisms rather than as established costs. The sixth category, soil natural-capital depreciation, is both the largest in principle and the least tractable in practice, because it requires a defensible estimate of the rate at which accumulating contamination reduces future productive potential, which in turn requires the calibrated dose–response relationship that Section 3.5 shows does not yet exist. This framework also clarifies why aggregate figures diverge so widely between studies, since analyses that count only flow costs will necessarily produce far smaller totals than those that attempt to capitalize the stock, and neither approach is wrong provided the choice is stated. We suggest that future economic assessments report costs disaggregated by this or a comparable taxonomy rather than as single headline totals, and that they state explicitly whether the counterfactual is a plastic-free system, a system with improved plastic management, or a system with no mulching at all, since these three counterfactuals generate materially different results. Adopting such conventions would allow the economic literature on agricultural plastics to become cumulative in a way that it currently is not.

**Table 7:** A structured accounting framework for the economic costs of microplastic contamination in agricultural systems, showing the appropriate valuation method and the current state of evidence for each category.

Cost category	Level at which cost arises	Appropriate valuation method	Current evidence status	Principal uncertainty
Yield-related revenue loss	Farm; aggregated to national and global	Physical shortfall × price, adjusted for demand elasticity and price transmission	Modelled only; global scoping estimates available	Uncalibrated dose–response; counterfactual specification
Film management and recovery	Farm	Partial budget; cost–benefit analysis with and without subsidy	Measured; field-survey based	Regional transferability; labor cost variation
Remediation and soil amendment	Farm; catchment	Cost of amendment plus foregone alternative use, net of yield recovery	Efficacy demonstrated at plot scale; costs not reported	No validated field-scale removal technology
Quality downgrading	Farm; first handler	Price differential between grades; hedonic analysis	Asserted; not quantified for MPs	No grading standard references particle burden
Market access and compliance	Exporter; smallholder aggregator	Certification, testing and audit cost; trade-flow displacement	Asserted; not quantified for MPs	Standards still in development; thresholds undefined
Soil natural-capital depreciation	Field asset; intergenerational	Capitalized loss of future productive potential; natural-capital accounting	Conceptual only	Requires calibrated long-run exposure–yield function

## 5 Food safety across the farm-to-fork continuum

### 5.1 Dietary exposure pathways and documented transfer

The uptake of microplastics into edible crops connects agricultural contamination to human dietary exposure, situating the problem within the broader domain of food safety and public health. Once particles enter edible tissues or adhere to produce surfaces, they become part of the food that reaches consumers, and the internalization of particles into crops has now been demonstrated experimentally under conditions relevant to agriculture (Yin et al., 2026; Wang et al., 2022). Trophic transfer along terrestrial food chains has likewise been demonstrated, with plastics moving from soil to detritivores and onward to higher consumers, establishing a pathway that culminates in humans at the top of the chain (Huerta Lwanga et al., 2017). This early field evidence of food-chain transfer remains a touchstone for subsequent work on dietary exposure and has shaped how the risk is conceptualized (Huerta Lwanga et al., 2017). Human exposure occurs mainly through ingestion, and microplastics have been associated in the toxicological literature with oxidative stress, inflammation, and other biological effects, although exposure estimates remain uncertain owing to methodological heterogeneity (Prata et al., 2020; Wright and Kelly, 2017). The co-transport of adsorbed heavy metals and organic pollutants compounds the concern, since microplastics can deliver these secondary contaminants into the diet alongside the particles themselves (Liu et al., 2024). Contamination is not confined to plant foods, because particles enter animal-derived products through livestock exposure, extending the issue across the whole diet (Huerta Lwanga et al., 2017; Prata et al., 2020). The absence of harmonized detection methods and agreed safety thresholds remains a fundamental obstacle to robust risk assessment and regulation (Prata et al., 2020; Ng et al., 2018). This uncertainty argues for precaution rather than complacency given the ubiquity of exposure (Wright and Kelly, 2017). Farming practices that reduce root injury and soil contamination therefore have a direct food-safety rationale in addition to an agronomic one (Yin et al., 2026). Food safety thus emerges as the pathway through which soil contamination acquires direct public-health salience. It also links the agricultural discussion to the wider human-health literature on plastics.

### 5.2 From detection to enforceable standards: the regulatory bottleneck

The food-safety implications of crop contamination will be realized economically through regulation rather than through toxicology alone, and the current absence of an enforceable regulatory architecture is therefore as consequential for producers as the underlying hazard. Three technical preconditions must be satisfied before any threshold can be set, and none is presently met. The first is a harmonized analytical protocol, because concentrations reported in edible tissue depend on extraction chemistry, minimum detectable particle size and identification technique to a degree that makes cross-study comparison unreliable, and the same methodological heterogeneity that prevents pooling of soil abundance data also prevents the construction of a defensible exposure distribution for dietary intake (Prata et al., 2020; Ng et al., 2018). The second is a mass-based rather than count-based metric, since toxicological reference values are conventionally expressed per unit body weight per day, whereas the microplastic literature overwhelmingly reports particle counts, and the conversion again requires unstated assumptions about size and density. The third is a resolved position on nanoplastics, which are the fraction most likely to cross biological barriers and to be internalized into edible tissue, yet are the fraction least reliably detected by the spectroscopic methods that dominate routine analysis, so a monitoring regime built on current capability would systematically miss the particles of greatest concern (Wang et al., 2022; Yin et al., 2026). Until these three conditions are met, no tolerable daily intake can be established, and in their absence regulators face a choice between inaction and precautionary measures that lack a quantitative basis, a dilemma that recent governance-oriented syntheses identify as the central obstacle to effective oversight of the soil-to-human continuum (Boctor et al., 2025). For producers, the practical consequence is a form of regulatory risk that is difficult to insure against, because standards may be introduced rapidly once methods mature, and compliance capacity will have to be built before the thresholds are known. This connects the food-safety discussion directly to the market-access costs identified in Section 4.4 and to the compliance-incidence argument in Section 4.5, since the burden of demonstrating conformity will fall on the same smallholders who are least able to absorb fixed costs. It also supplies a further argument for prioritizing source reduction, because a lower particle burden is the only strategy that remains robust regardless of where thresholds are eventually set. Farming practices that reduce root injury and limit contamination therefore have an anticipatory regulatory rationale in addition to their agronomic and public-health one (Yin et al., 2026).

## 6 Supply chain resilience and systemic vulnerability

### 6.1 The plasticulture dependency and the resilience paradox

Viewed at the level of the whole food system, microplastic contamination represents a slow-onset stressor that gradually erodes the resilience on which stable food supply depends. Modern agriculture has become deeply dependent on plastics for productivity, water efficiency, and post-harvest protection, so that plasticulture is now embedded in the operating model of intensive food production worldwide (Sattar et al., 2025; Gontard, 2025). This dependency generates a resilience paradox, because the material that enhances short-term productivity simultaneously degrades the long-term productive capacity of soils through cumulative contamination (Gontard, 2025). Unlike acute shocks such as droughts or price spikes, chronic microplastic accumulation acts gradually and often invisibly, which means its systemic costs accrue before they are recognized and are difficult to reverse once realized (Sattar et al., 2025; Gontard, 2025). The contamination is also transmitted through the supply chain itself, since plastics enter at multiple nodes from field inputs to packaging, and each stage can add to or redistribute the particle burden (Gontard, 2025). A recent perspective argued for naming and framing the plastic-particle footprint of the agri-food sector precisely so that these long-term, cross-stage impacts can be weighed against short-term benefits in strategic decision-making (Gontard, 2025). Concentration of production in contamination-prone regions and reliance on globally traded inputs further couple local contamination to distant markets, so that soil degradation in one region can propagate as supply risk elsewhere (Zhu et al., 2025; FAO, 2025). Because the productivity losses modelled at the global scale fall on the same staple crops that anchor food security, the systemic exposure is concentrated precisely where resilience matters most (Zhu et al., 2025). Framing microplastic contamination as a systemic vulnerability rather than a local nuisance reorients attention toward structural rather than merely technical responses (Gontard, 2025). **Figure 4**, proposed later, is intended to depict the cascade from particle to plant to profit to system. The systemic lens is what distinguishes contamination as a strategic rather than incidental concern. It also clarifies why governance, considered next, is indispensable.

**Figure 4:**
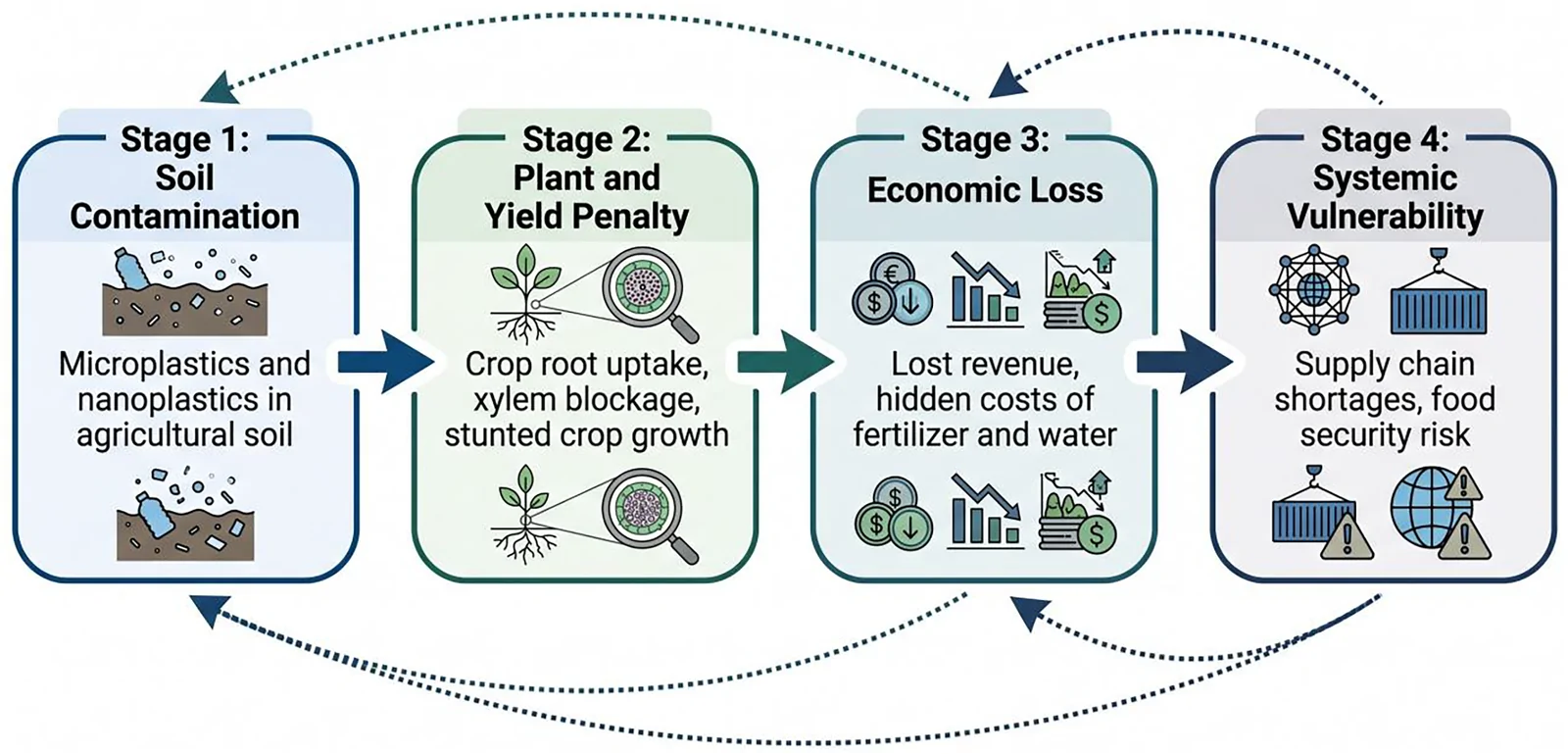
The particle-to-plant-to-profit-to-system cascade. Design as a horizontal flow diagram with four linked stages: soil contamination, plant/yield penalty, economic loss (revenue and hidden costs), and supply-chain/systemic vulnerability, with feedback arrows. This image visually anchors the review’s integrative thesis.

### 6.2 Governance, standards, and circular-economy levers

Because microplastic contamination crosses jurisdictional and sectoral boundaries, its management increasingly depends on governance instruments that operate at national and international scales alongside farm-level practice. The Food and Agriculture Organization has responded by developing a Voluntary Code of Conduct on the Sustainable Use and Management of Plastics in Agriculture, published in provisional form in early 2025, which provides a framework for reducing and better managing agricultural plastics (FAO, 2025). This instrument is significant because it explicitly addresses agricultural plastics as a distinct policy category rather than subsuming them under general plastics regulation (FAO, 2025). At the conceptual level, proposals to account for the plastic-particle footprint of the agri-food sector would provide the metrics needed to hold actors accountable across the supply chain (Gontard, 2025). Circular-economy thinking is central to these efforts, emphasizing reduced reliance on finite, contamination-generating materials and closed-loop handling of resources to strengthen resilience (Gontard, 2025). Effective governance must engage farmers, industry, scientists, and regulators simultaneously, and must preserve traditional and nature-based practices that enhance resilience rather than displacing them (Gontard, 2025; FAO, 2025). Source-directed measures such as improved film recovery, thickness standards, and better sludge treatment are most effective when embedded in supportive policy and market structures (Sattar et al., 2025; Yang et al., 2022). The alignment of emerging instruments with the scientific evidence reviewed here is a precondition for proportionate action, since policy that outpaces or lags the science risks being either burdensome or ineffective (Gontard, 2025; Zhu et al., 2025). Monitoring frameworks that track polymers, additives, and contamination across the food system are needed to build the evidence base for accountability (Gontard, 2025). Governance thus supplies the systemic complement to the technical mitigation measures considered next. Its development remains a work in progress that will shape the trajectory of contamination. Coordinating these instruments internationally is among the central challenges ahead.

### 6.3 Characterizing systemic exposure: concentration, correlation, and irreversibility

Describing microplastic contamination as a systemic risk is only useful if the properties that make it systemic can be specified, and four such properties can be identified from the evidence assembled in this review. The first is concentration of exposure, because contamination is not distributed uniformly across production but clusters in intensively managed systems, so that a small number of high-output regions account for a disproportionate share of the global burden, and the strongly right-skewed distribution evident in national survey data is the statistical signature of this clustering (Chen et al., 2022; Yu et al., 2023). Systems whose output is concentrated in few locations transmit local degradation into global supply far more efficiently than diversified systems, which is why the same tonnage of loss has different systemic significance depending on where it occurs. The second property is positive correlation with other stressors, and this is the least appreciated feature of the problem, because plasticulture is most intensively deployed precisely where water is scarce and the growing season is short, which means that the regions carrying the heaviest microplastic burdens are also those most exposed to drought and heat stress. Microplastic pressure is therefore not an independent risk that diversifies away against climate risk but a correlated one that compounds it, and correlated risks are the class that portfolio logic cannot absorb. The third property is irreversibility over decision-relevant timescales, since no validated field-scale removal technology exists, weathering increases rather than decreases the reactivity of the accumulated stock, and the twenty-three-year biosolid record shows accumulation proceeding across an entire management career (Adhikari et al., 2024; Ren et al., 2021; Wu et al., 2024). A stressor that cannot be reversed converts a management problem into a constraint on the feasible set of future production systems. The fourth property is invisibility within existing accounting and monitoring systems, demonstrated quantitatively by the finding that environmental and external benefits constitute under one percent of the accounted benefits in farm-level cost–benefit analysis of mulch management, which means the signal is not weak but absent from the ledger that guides decisions (Hao et al., 2024). Taken together, these four properties define a risk profile that is concentrated, correlated, irreversible and unmeasured, which is precisely the combination that produces surprise in food systems rather than gradual adaptation. Framing the problem in these terms also indicates where systemic intervention should be directed, namely at monitoring in high-concentration regions, at the correlation with water-scarce production, and at bringing the externality into the accounting frame, rather than at uniform measures applied across all cropland. **Figure 4** depicts the resulting cascade from particle to plant to profit to system.

## 7 Mitigation and remediation pathways

### 7.1 Source reduction and material substitution

Global distribution of microplastics have been reported in different agricultural soil as shown in **Figure 5**. The most robust research response to microplastic contamination follows the environmental hierarchy of preventing inputs before attempting to remove particles already present in the soil. Source reduction begins with improving the recovery of agricultural films through thickness standards, mechanized collection, and recycling schemes that raise the currently low rates of film retrieval (Sattar et al., 2025; Qi et al., 2020). Substituting conventional polyethylene mulch with biodegradable alternatives is frequently proposed, yet the evidence urges caution, because many biodegradable films require industrial composting conditions rarely met in soil and can fragment into persistent biodegradable microplastics that themselves affect plants and microbes (Fan et al., 2022; Yang et al., 2022). Some studies indeed report that biodegradable mulches generate microplastics faster than conventional films under field conditions, so substitution must be evaluated empirically rather than assumed beneficial (Yang et al., 2022; Fan et al., 2022). Upstream measures such as microbead bans, improved wastewater and sludge treatment, and stricter control of compost feedstock can substantially reduce the microplastic load delivered through organic amendments (Sattar et al., 2025; Corradini et al., 2019). Farmer awareness and capacity-building are repeatedly identified as important, since understanding of the long-term consequences of plastic residues remains limited and behavioral change is a precondition for source reduction (FAO, 2025; Gontard, 2025). Selecting irrigation methods that minimize particle deposition and downward transport, such as drip in preference to flood irrigation, offers a further practical lever (Garfansa et al., 2025; Xing et al., 2021). Practices that reduce root injury during transplanting and cultivation can additionally limit the uptake of particles into edible tissues, linking agronomic technique to food safety (Yin et al., 2026). Source reduction is generally more cost-effective and durable than downstream remediation, which reinforces its priority in any management strategy (Gontard, 2025). **Table 5** summarizes mitigation and remediation options alongside their mechanisms and limitations. Prevention, in short, remains the foundation of effective control.

**Figure 5:**
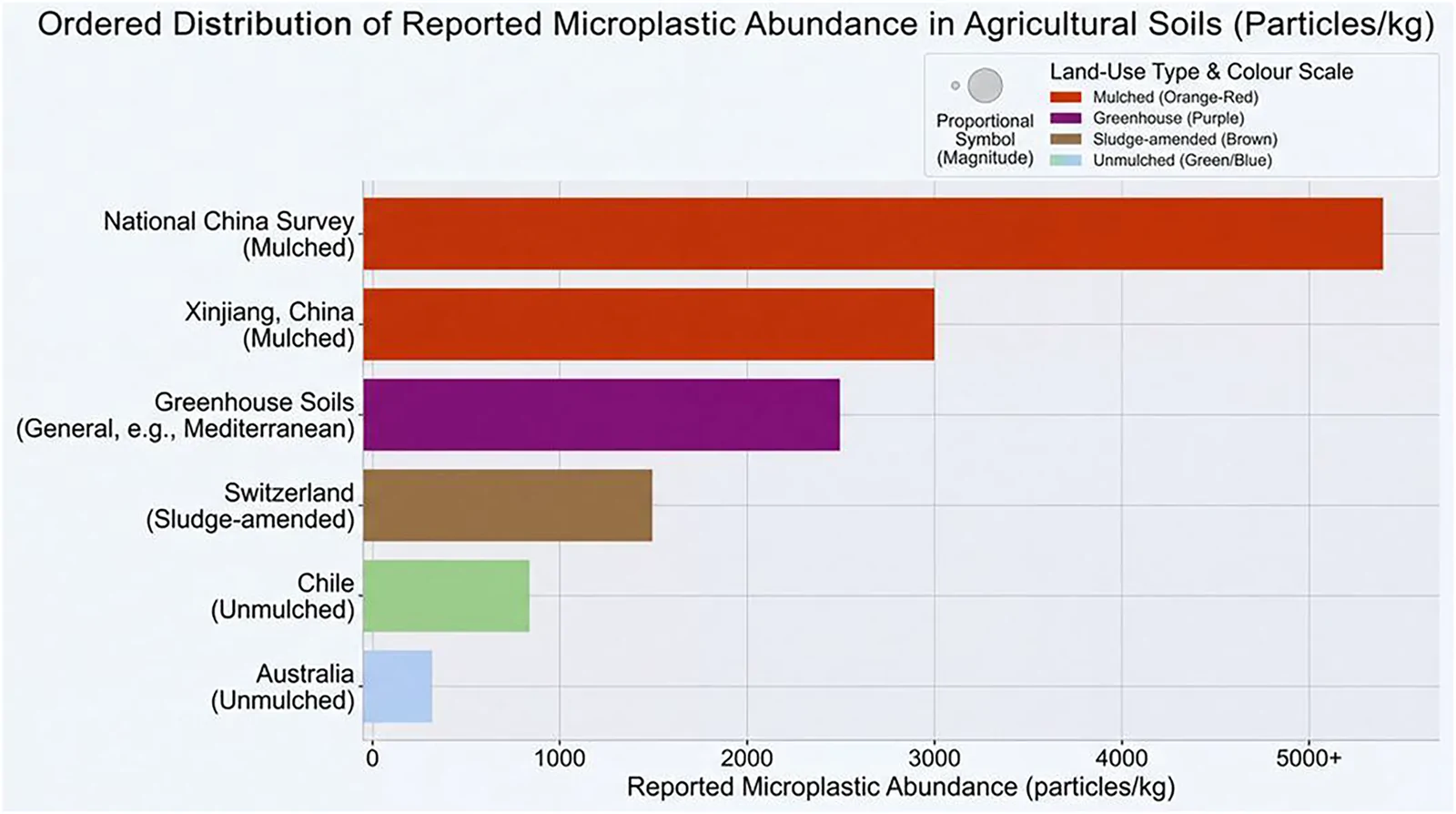
Distribution of reported microplastic abundance in agricultural soils. The image is an ordered bar chart of mean reported abundance, expressed in particles per kilogram of dry soil, for each of the study locations compiled in **Table 4**, namely Xinjiang (mulched cotton), the national Chinese farmland survey, Chile (sludge-amended), Australia (biosolid-amended) and Switzerland (floodplain soils). Bars are ordered from highest to lowest reported abundance, error bars denote the dispersion reported in the original study where available, and locations for which the source reports only a qualitative range are shown without a bar and annotated as not quantified. Because extraction efficiency and minimum detectable particle size differ among the source studies, the chart should be read as a comparison of reported values rather than of harmonized measurements.

### 7.2 Soil amendments and emerging remediation approaches

Where microplastics are already present, a growing toolbox of remediation approaches seeks to reduce their abundance or ameliorate their effects, although none yet offers a complete solution at field scale. Biochar has attracted particular attention as a soil amendment, because its porous structure and reactive surface can adsorb particles, stabilize soil aggregates, improve moisture retention, regulate pH, and support beneficial microbial communities in contaminated soils (Awet et al., 2018; Wu et al., 2024). A controlled study reported that biochar mitigated the negative effects of microplastics on sugarcane growth by altering soil nutrients and reshaping the microbial community, illustrating a practical remediation benefit (Wu et al., 2024). Earlier experimental work similarly showed that biochar amendment could partially restore enzyme activities and microbial biomass carbon suppressed by polymer particles, indicating a functional as well as structural benefit (Awet et al., 2018). Biological degradation offers a complementary route, exploiting the capacity of soil microorganisms and the plastisphere to slowly modify polymer structure, although rates are slow and highly polymer-specific (Rillig and Lehmann, 2020; Ma et al., 2023). Soil fauna may contribute modestly to particle redistribution and to the buffering of microplastic stress on soil communities, positioning bioturbation as a factor in both dispersal and amelioration (Rillig et al., 2017; Huerta Lwanga et al., 2016). These approaches face important limitations, including slow degradation rates, incomplete removal, potential secondary effects of amendments on particular soil types, and a general lack of field validation at scale (Wu et al., 2024; Fan et al., 2022). The most promising strategies are likely to combine prevention, soil amendment, and biological processes within an integrated management framework rather than relying on any single technique (Gontard, 2025). Careful evaluation is needed to ensure that remediation does not introduce new trade-offs, since biodegradable substitutes and some amendments carry their own risks (Fan et al., 2022). **Figure 6**, proposed later, is intended to present such an integrated decision framework. Remediation therefore complements but cannot substitute for source control. Its maturation into field-ready practice is a priority for the coming decade.

**Figure 6:**
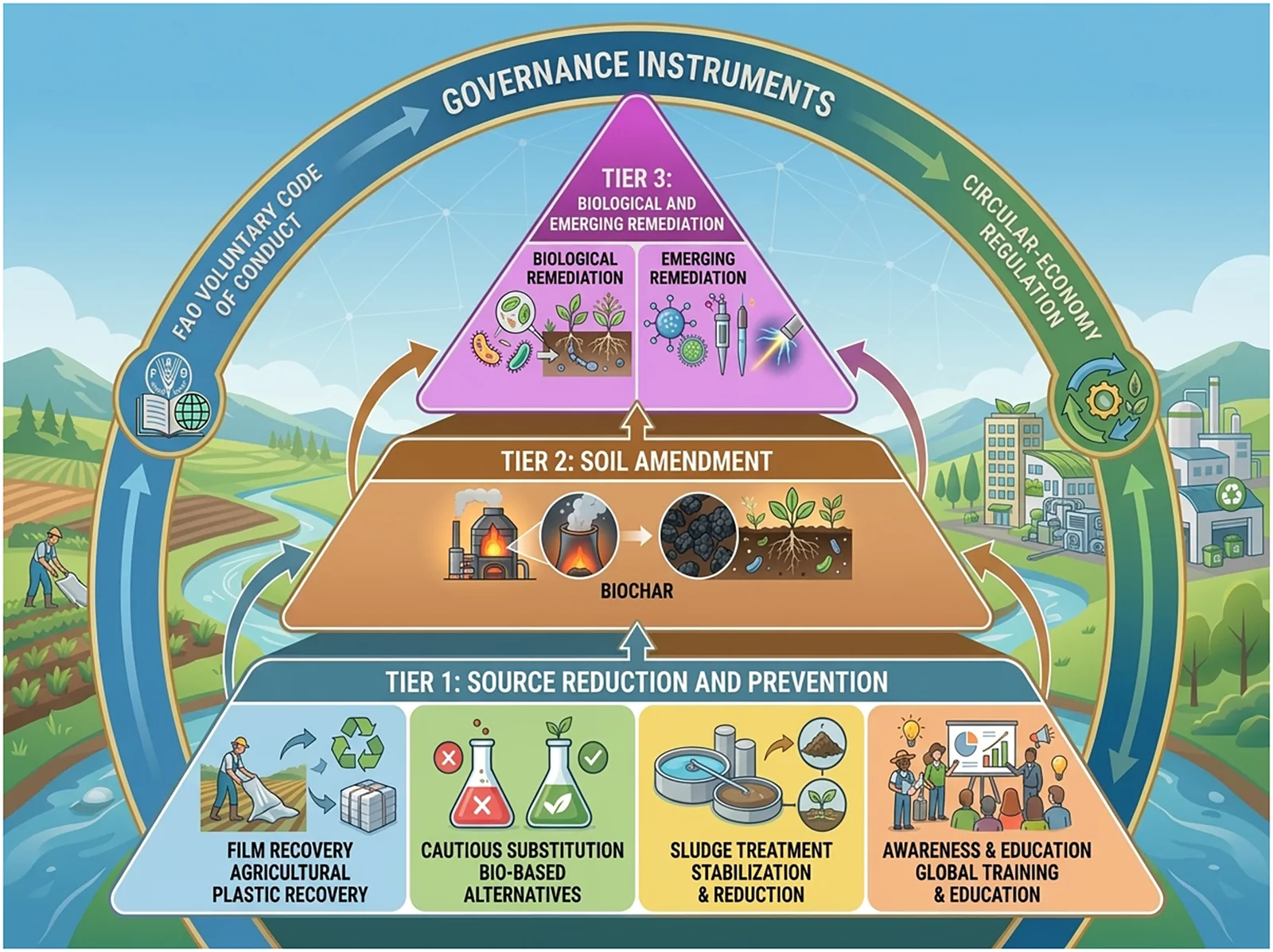
Integrated mitigation and remediation decision framework. A tiered pyramid or decision tree placing source reduction and prevention at the base (film recovery, cautious substitution, sludge treatment, awareness), soil amendment in the middle (biochar), and biological and emerging remediation at the top, with governance instruments (FAO Voluntary Code of Conduct, circular-economy regulation) as an enclosing band.

### 7.3 Cost-effectiveness, sequencing, and adoption barriers

Mitigation options are conventionally presented as a menu, but the economic evidence assembled in Section 4.3 permits something more useful, namely a provisional ordering by cost-effectiveness that can guide sequencing under budget constraints. The first tier comprises measures that are privately profitable and therefore require only enabling infrastructure rather than sustained transfers, and the clearest example is the shift to thicker, mechanically recoverable film, which delivered a net benefit exceeding that of conventional film by approximately CNY 253.8 per hectare while simultaneously improving recoverability and generating recyclable material (Hao et al., 2024). Because this option pays for itself, the binding constraint is not price but the availability of collection and recycling infrastructure and grower awareness, which makes it the most efficient first target for public expenditure. The second tier comprises measures that are inexpensive but whose benefit is contingent on context, including irrigation method selection where drip infrastructure already exists, tighter control of compost feedstock, improved sludge processing, and cultivation practices that limit root injury and thereby reduce translocation into edible tissue (Xing et al., 2021; Sattar et al., 2025; Yin et al., 2026). The third tier comprises measures with demonstrated agronomic benefit but unquantified field-scale cost, of which biochar amendment is the leading example, having been shown to mitigate microplastic effects on sugarcane growth and to partially restore enzyme activities and microbial biomass carbon, but never costed at operational scale against the yield recovery it delivers (Wu et al., 2024; Awet et al., 2018). The fourth tier comprises measures that are currently unprofitable, dependent on subsidy, and of contested environmental benefit, and biodegradable film substitution falls squarely into this category, since it reduced net benefit to approximately CNY 2,244.6 per hectare in the Chinese analysis, showed profitability highly sensitive to price discounts in the United States analysis, faces a documented willingness-to-pay shortfall among growers, and may fragment into microplastics faster than the conventional film it replaces (Hao et al., 2024; Velandia et al., 2020a, 2020; Fan et al., 2022; Yang et al., 2022). This ordering is provisional and regionally contingent, since labor costs, film prices, disposal fees and subsidy regimes vary substantially, and the underlying analyses derive from China and the United States rather than from the regions where the evidence base is thinnest. It nonetheless supports a clear practical conclusion, namely that the current policy emphasis on material substitution inverts the cost-effectiveness ordering, directing scarce public funds toward the least efficient tier while the most efficient tier remains constrained by infrastructure that is comparatively cheap to provide. **Table 8** accordingly reports mitigation options together with their economic status as well as their mechanisms and limitations, and **Figure 6** presents the integrated decision framework.

**Table 8:** Mitigation and remediation strategies for microplastic contamination in agricultural systems: mechanisms, economic status and limitations.

Strategy	Mechanism	Economic status	Key limitations	Source
Film recovery & thickness standards	Reduces residual macroplastic that fragments to MPs	Privately profitable; +CNY 253.8/ha vs conventional film	Low recovery of thin films; labor cost	Qi et al. (2020); Sattar et al. (2025); Hao et al. (2024)
Biodegradable mulch substitution	Replaces persistent PE with degradable polymers	Unprofitable without subsidy; CNY 2,244.6/ha; WTP below market price	May fragment faster; incomplete soil mineralization	Yang et al. (2022); Fan et al. (2022); Velandia et al. (2020a)
Improved sludge/compost treatment	Lowers MP load in amendments	Cost borne off-farm; not quantified for MPs	Cost; nutrient-recycling trade-off	Corradini et al. (2019); Sattar et al. (2025)
Biochar amendment	Adsorbs particles; stabilizes aggregates; supports microbes	Agronomic benefit demonstrated; field-scale cost not reported	Field validation limited; context-dependent	Wu et al. (2024); Awet et al. (2018)
Drip vs flood irrigation	Reduces particle deposition and downward transport	Low marginal cost where infrastructure exists; high if new	Infrastructure cost; not always feasible	Garfansa et al. (2025); Xing et al. (2021)
Root-injury-minimizing cultivation	Limits wound-mediated uptake into edible organs	Near-zero direct cost; labor reallocation only	Efficacy not yet tested at field scale	Yin et al. (2026)
Governance & source policy	Reduces inputs system-wide	Administrative cost; externality currently ~0.9% of accounted benefit	Requires coordination and enforcement	FAO (2025); Gontard (2025); Hao et al. (2024)

## 8 Knowledge gaps and future research priorities

The gaps that matter most for this review are not the generic ones that recur across the microplastic literature, such as analytical standardization and nanoplastic detection, which are real but are already the subject of dedicated methodological programmes and are only indirectly connected to the productivity, economic and supply-chain questions addressed here. We therefore organize priorities around the three links in the causal chain that the review traces, and we specify in each case what would have to be measured rather than merely noting that uncertainty exists. The first cluster concerns the productivity link, where the central requirement is a set of field-realistic exposure–yield functions for major staples, obtained from multi-season trials conducted at contamination levels matching measured field abundances rather than at the mass fractions that dominate current experiments. Such trials must report exposure in both particle counts and mass fractions with full characterization of size distribution, morphology and polymer identity, since it is the absence of this dual reporting that currently prevents the field survey and the phytotoxicity literatures from being combined (Chen et al., 2022; Sun H. et al., 2024). They must also run for multiple seasons, because the effect of interest accumulates over decades while the evidence base is dominated by single-season observations, and they must include co-contaminated soils, given the systematic exacerbation demonstrated under co-exposure (Adhikari et al., 2024; Wang et al., 2024). A related priority is to resolve whether harvest index declines under exposure, since the trait-level finding that reproductive organs are inhibited more strongly than vegetative ones implies that biomass-based extrapolations understate yield loss, and this single parameter substantially affects every global estimate currently in circulation (Sun H. et al., 2024; Zhu et al., 2025).

The second cluster concerns the economic link, where the requirement is to move from scoping calculations to decision-grade estimates, and the necessary components can be specified precisely. Spatially explicit exposure surfaces are needed, because loss estimates currently apply a global mean effect size to a global production base while contamination is strongly clustered, and this mismatch is a larger source of error than uncertainty in the effect size itself. Loss models must incorporate demand elasticity and price transmission so that producer revenue loss, consumer welfare loss and international transfer are reported separately rather than conflated, and they must state their counterfactual explicitly, distinguishing a plastic-free system from an improved-management system and from a no-mulching system, since these generate materially different results (Ma et al., 2018; Hao et al., 2024). Farm-level cost–benefit analyses of the kind now available for northern China and the United States are required for other major plasticulture regions, because the finding that environmental externalities account for under one percent of accounted benefit is the most policy-relevant quantitative result in this literature and its generality is unknown (Hao et al., 2024; Velandia et al., 2020b). Methods for capitalizing soil natural-capital depreciation must be developed and tested, since Section 4.6 identifies this as simultaneously the largest cost category in principle and the only one with no empirical basis at all. Finally, the costs asserted for quality downgrading, market access restriction and compliance require primary quantification, because they are currently repeated across the literature without a single published estimate specific to microplastics.

The third cluster concerns the systemic and governance link, where the priority is to characterize the four properties identified in Section 6.3 empirically rather than conceptually. Production-weighted exposure indices would establish how much of global staple output originates in high-burden regions; correlation analyses between contamination intensity and climatic water deficit would test the hypothesis that microplastic and climate stress are positively rather than independently distributed; and irreversibility should be quantified through long-term monitoring of whether abandonment of mulching in previously intensive systems produces measurable decline in soil particle burden over decadal timescales. Governance research should evaluate instruments against the cost-effectiveness ordering developed in Section 7.3 rather than against stated environmental ambition, and should test whether subsidy reallocation from material substitution toward recovery infrastructure delivers greater abatement per unit of public expenditure (FAO, 2025; Gontard, 2025). Cutting across all three clusters is a geographical imbalance in the evidence base that is severe enough to constitute a substantive limitation of this review and of every synthesis preceding it. A review of terrestrial microplastic contamination studies found that Asian countries accounted for approximately 60 percent of published work and European countries for approximately 29 percent, while African and North American countries contributed approximately 4 percent each and Latin America approximately 3 percent (Sa’adu and Farsang, 2023). The African position is more constrained still, with a recent assessment identifying only about six studies of microplastic contamination and effects in African terrestrial ecosystems, distributed across just four countries (Tchiechoua and Rillig, 2025). A synthesis of 105 field datasets reached the same conclusion from the opposite direction, noting that the dominance of East and South Asian data, and Chinese data in particular, constrains the robustness of global analysis and calling for expanded work in the Americas, Australia and Africa (Roy et al., 2025). Because the conclusions of this review, including the abundance ranges in **Table 4**, the effect sizes in **Table 5** and the farm-level economics in **Table 6**, rest disproportionately on Chinese and other East Asian evidence, their transferability to South Asian smallholder systems, sub-Saharan African rainfed agriculture and Latin American production landscapes should not be assumed. This is not a peripheral caveat, because these regions differ in the very variables that govern outcomes, including mulching intensity, biosolid use, irrigation method, soil temperature regime, farm scale and regulatory capacity, and because smallholder-dominated systems that rely on crop-residue rather than plastic mulching may face a source profile in which amendments, irrigation and deposition dominate rather than film (Tchiechoua and Rillig, 2025). Generating primary field and economic data in these regions is therefore the single highest-value contribution the field could make in the coming decade, and it should precede rather than follow further global extrapolation.

## 9 Conclusion

Microplastic contamination of agricultural systems has emerged, within a remarkably short span of research, as a credible and multidimensional threat to the productivity, economics, and resilience of global food production. The evidence reviewed here demonstrates that agricultural soils function as major reservoirs of plastic, receiving continuous inputs from mulch films, organic amendments, irrigation, coated inputs, and atmospheric deposition, and that these particles transform over time into smaller, more reactive, and more mobile forms. Within the soil, microplastics reshape physical structure, hydrology, microbial communities, enzyme activity, and nutrient cycling, while also disturbing the soil fauna that engineer fertility. These soil-level disturbances propagate into the plant, where microplastics inhibit germination, impair root architecture, suppress photosynthesis, disrupt nutrient acquisition, and, in their smallest forms, penetrate tissues and accumulate in edible organs. A defining contribution of recent research has been to quantify the aggregate consequence of these effects, with global modelling suggesting that microplastic-induced photosynthetic suppression alone may cost the world hundreds of millions of tons of staple crop production each year. Translated into economic terms, these losses, together with hidden costs of remediation, quality downgrading, and constrained market access, constitute a measurable and growing burden that is currently underestimated in conventional accounting. Viewed systemically, the dependence of modern agriculture on plastics generates a resilience paradox in which a productivity aid becomes a slow-onset stressor eroding the productive base, transmitted through and amplified across the supply chain. Mitigation is nonetheless feasible, and the balance of evidence favors prevention through source reduction and careful material substitution, complemented by biochar amendment and reinforced by emerging governance instruments such as the FAO Voluntary Code of Conduct and circular-economy regulation. Realizing this potential requires resolving persistent uncertainties around field-realistic exposure, nanoplastic behavior, translocation into food, and economic magnitude through coordinated interdisciplinary research. The central message of this review is that microplastic contamination should be reframed from an ecotoxicological curiosity into a strategic risk to food systems, deserving of proportionate scientific, economic, and policy attention. Acting on that recognition promptly offers the best prospect of safeguarding soil health, crop productivity, and the resilience of the food supply for future generations. Two qualifications should accompany that message, and stating them strengthens rather than weakens it. The first is epistemic, in that the global loss figures now circulating are scoping estimates constructed through a chain of extrapolations whose net bias is undetermined, and the case for action rests less on their precise magnitude than on the convergence of independent evidence that the direction of effect is negative and the accumulation irreversible. The second is geographical, in that the evidence supporting this synthesis derives overwhelmingly from East Asian and European field data, so that its quantitative conclusions should be treated as regionally grounded rather than universal until comparable data exist for South Asia, sub-Saharan Africa and Latin America. What the evidence does support without qualification is a reordering of priorities: bringing the contamination externality into the accounting frameworks that currently value it at under one percent of measured benefit, directing mitigation expenditure first toward the recovery measures that are already privately profitable, and generating the field-realistic exposure–yield data without which no economic estimate can be more than indicative.
